# Estimating photosynthetic traits from reflectance spectra: A synthesis of spectral indices, numerical inversion, and partial least square regression

**DOI:** 10.1111/pce.13718

**Published:** 2020-02-27

**Authors:** Peng Fu, Katherine Meacham‐Hensold, Kaiyu Guan, Jin Wu, Carl Bernacchi

**Affiliations:** ^1^ Carl R. Woese Institute for Genomic Biology University of Illinois at Urbana‐Champaign Urbana Illinois; ^2^ Department of Plant Biology University of Illinois at Urbana‐Champaign Urbana Illinois; ^3^ Department of Natural Resources and Environmental Sciences University of Illinois at Urbana Champaign Urbana Illinois; ^4^ National Center for Supercomputing Applications University of Illinois at Urbana Champaign Urbana Illinois; ^5^ School of Biological Sciences The University of Hong Kong Pokfulam Hong Kong; ^6^ USDA‐ARS Global Change and Photosynthesis Research Unit University of Illinois at Urbana‐Champaign Urbana Illinois

**Keywords:** earth system models, global carbon cycles, high‐throughput mapping, hyperspectral imaging, machine learning, photosynthesis, plant breeding

## Abstract

The lack of efficient means to accurately infer photosynthetic traits constrains understanding global land carbon fluxes and improving photosynthetic pathways to increase crop yield. Here, we investigated whether a hyperspectral imaging camera mounted on a mobile platform could provide the capability to help resolve these challenges, focusing on three main approaches, that is, reflectance spectra‐, spectral indices‐, and numerical model inversions‐based partial least square regression (PLSR) to estimate photosynthetic traits from canopy hyperspectral reflectance for 11 tobacco cultivars. Results showed that PLSR with inputs of reflectance spectra or spectral indices yielded an *R* ^2^ of ~0.8 for predicting *V*_*cmax*_ and *J*_*max*_, higher than an *R* ^2^ of ~0.6 provided by PLSR of numerical inversions. Compared with PLSR of reflectance spectra, PLSR with spectral indices exhibited a better performance for predicting *V*_*cmax*_ (*R* ^2^ = 0.84 ± 0.02, *RMSE* = 33.8 ± 2.2 μmol m^−2^ s^−1^) while a similar performance for *J*_*max*_ (*R* ^2^ = 0.80 ± 0.03, *RMSE* = 22.6 ± 1.6 μmol m^−2^ s^−1^). Further analysis on spectral resampling revealed that *V*_*cmax*_ and *J*_*max*_ could be predicted with ~10 spectral bands at a spectral resolution of less than 14.7 nm. These results have important implications for improving photosynthetic pathways and mapping of photosynthesis across scales.

## Abstract 2

Photosynthetic capacities can be predicted with a *R* ^2^ of ~0.8 using plot‐level hyperspectral reflectance and partial least squares regression. About 10 spectral bands with the spectral resolution less than 14.7 nm are enough for accurately estimating photosynthetic capacities.

## 1 INTRODUCTION

Photosynthetic traits of vegetation canopies are important parameters of process‐based Earth system models to understand global carbon cycles (Croft et al., 2017; Rogers, 2014; Schaefer et al., 2012). However, the lack of spatially and temporally continuous information on photosynthetic traits for these Earth system models results in a large uncertainty to account for carbon sinks, sources, and exchange between the atmosphere and the terrestrial biosphere (Rogers, 2014). In addition, accurate characterization of photosynthetic rates holds crucial merits to redesign photosynthesis pathways to improve crop yield (Long & Ort, 2010; Ort et al., 2015; Ray et al., 2012; Tilman et al., 2011). At present, improving photosynthesis remains a source of untapped potential because photosynthesis is far from its biological limits (Long et al., 2006; Zhu et al., 2008). The selection of new cultivars requires linkages of genotypes to phenotypes in a given environment, yet it has not achieved in a high‐throughput manner, becoming one of the major bottlenecks in plant breeding (Cabrera‐Bosquet et al., 2012; Furbank & Tester, 2011). To this end, technical advances in high‐throughput characterization of photosynthetic traits are highly needed as part of solutions to the global food security problem and are critical for a deep understanding of global environmental change.

The maximum potential for photosynthesis in C3 crops is largely determined by the maximum rate of carboxylation (*V*_*cmax*_) and the maximum rate of electron transport (*J*_*max*_; Long & Bernacchi, 2003). The two variables, together with a widely used biochemical model (Farquhar et al., 1980), can be used to understand crop photosynthetic performance at leaf to ecosystem levels (Thum et al., 2007; van der Tol et al., 2009; Zhang et al., 2014). Traditionally, *V*_*cmax*_ and *J*_*max*_ are measured in vivo through gas exchange systems (e.g., LI‐6800 Portable Photosynthesis System), which is costly and time‐consuming. Recently, studies have shown that reflectance spectra measured by a high‐spectral‐resolution spectroradiometer can capture *V*_*cmax*_ and *J*_*max*_ variations across species and temperature regimes (Ainsworth et al., 2014; Heckmann et al., 2017; Serbin et al., 2012; Serbin et al., 2015; Silva‐Perez et al., 2018). Despite success of leaf‐level estimations of photosynthetic capacities using point‐based spectral analysis, few studies used hyperspectral imaging (HSI) technique for high‐throughput estimations of photosynthesis beyond leaf level (Serbin et al., 2015). In addition to provide a large number of samples (orders of magnitude more pixels than a point‐based sensor), HSI may reveal significant variability in photosynthetic traits of interest across a leaf, within a plant, from plant to plants, among genetically distinct lines, or/and over large geographic areas. Despite these advantages of HSI, significant challenges in canopy‐scale analysis exist. First, the growing availability of hyperspectral measurements from on‐site, close‐range, and remote platforms results in accumulation of hyperspectral data in both spatial and temporal domains. The collection of sensor‐based photosynthetic measurements already shifted the research focus from data procurement to data mining of abundant spectral information (Araus & Cairns, 2014). However, data mining of hyperspectral data is limited by the number of ground‐truth photosynthetic measurements (in particular, the number of samples is less than the dimension of hyperspectral data). Second, spectral reflectance captured by a hyperspectral sensor at the canopy level is more complex and composited by multisource variability, such as those associated with plant geometry and architecture, leaf scattering properties, and background soil (Jay et al., 2017; Mohd Asaari et al., 2018). Thus, spurious spectral variations are introduced in the recorded signals, blurring spectral signatures associated with target photosynthetic traits.

To overcome these issues, numerous machine learning algorithms have been proposed to infer photosynthetic traits from the unique profile of reflectance values in the visible (400–700 nm), near‐infrared (700–1,200 nm), and shortwave infrared regions (1200–2,500 nm; Heckmann et al., 2017; Serbin et al., 2012; Serbin et al., 2015; Yendrek et al., 2017). These spectral regions are generally associated with leaf properties. For example, the reflectance spectrum in the visible region is mainly dominated by light absorption of leaf pigments (Huete, 2004), whereas reflectance in the shortwave region is related to water absorption and dry matter (Ceccato et al., 2001; Jacquemoud & Baret, 1990). Given these reflectance‐leaf characteristics associations, machine learning algorithms such as partial least square regression (PLSR; Geladi & Kowalski, 1986; Wold et al., 2001) and artificial neural network regression (Specht, 1991) are widely used for analysing hyperspectral data because of their ability to deal with irrelevant spectral bands and band collinearity, also known as the curse of dimensionality of data (Thenkabail et al., 2013). The understanding of the physiological mechanism for correlating reflectance spectra with photosynthetic variables, however, remained unsolved using complex machine learning algorithms (Fu et al., 2019). Alternatively, vegetation indices (VIs) such as normalized difference VI, photochemical reflectance index, and chlorophyll index have also been used to reveal photosynthetic productivity (Ainsworth et al., 2014; Drolet et al., 2005; DuBois et al., 2018; Gamon et al., 1992; Muraoka et al., 2013). However, the potential of these VIs and their best band combinations have seldomly been explored to map photosynthesis at the canopy level.

Inversion of radiative transfer models (RTMs), which allows for the simulation of reflectance at arbitrary viewing and illumination angles (Jacquemoud & Baret, 1990; Roosjen et al., 2018; Verhoef, 1984), appears as a promising approach to infer photosynthetic traits from hyperspectral reflectance among different cultivars. RTMs such as PROSPECT (Jacquemoud & Baret, 1990) and PROSAIL (Jacquemoud et al., 2009) have been used to characterize structural and biochemical parameters, for example, leaf area index (LAI), chlorophyll content, and dry matter content (Clevers & Kooistra, 2012; Darvishzadeh et al., 2008; Duan et al., 2014; Si et al., 2012). Jay et al. (2017) showed that the PROSAIL model, evaluated over 14 sugar beet cultivars, could well estimate LAI and chlorophyll content with root mean square error (RMSE) ≤10%. Given the close relationship between leaf characteristics (e.g., leaf pigments, structure, water, and dry mass content) and photosynthetic traits (Ceccato et al., 2001; Jacquemoud & Baret, 1990; Lobato et al., 2010), the reduction of hyperspectral reflectance into several meaningful biophysical parameters through RTMs may thus help identify subtle differences in photosynthetic traits among different cultivars.

In this study, three different approaches using PLSR with inputs of reflectance spectra, spectral indices, and RTM‐derived crop traits, respectively, were synthesized and compared relative with their ability to reveal photosynthetic differences among crop cultivars. Data analysis was based on millimetre hyperspectral imagery collected from a ground‐based phenotyping platform. Further analysis was also performed to evaluate the predictive performance of PLSR with reflectance spectra as inputs across a series of spectral resolutions to understand whether sensors with multispectral bands are suitable for estimating photosynthetic rates.

## 2 MATERIALS AND METHODS

### 2.1 Experimental design

**Figure 1:**
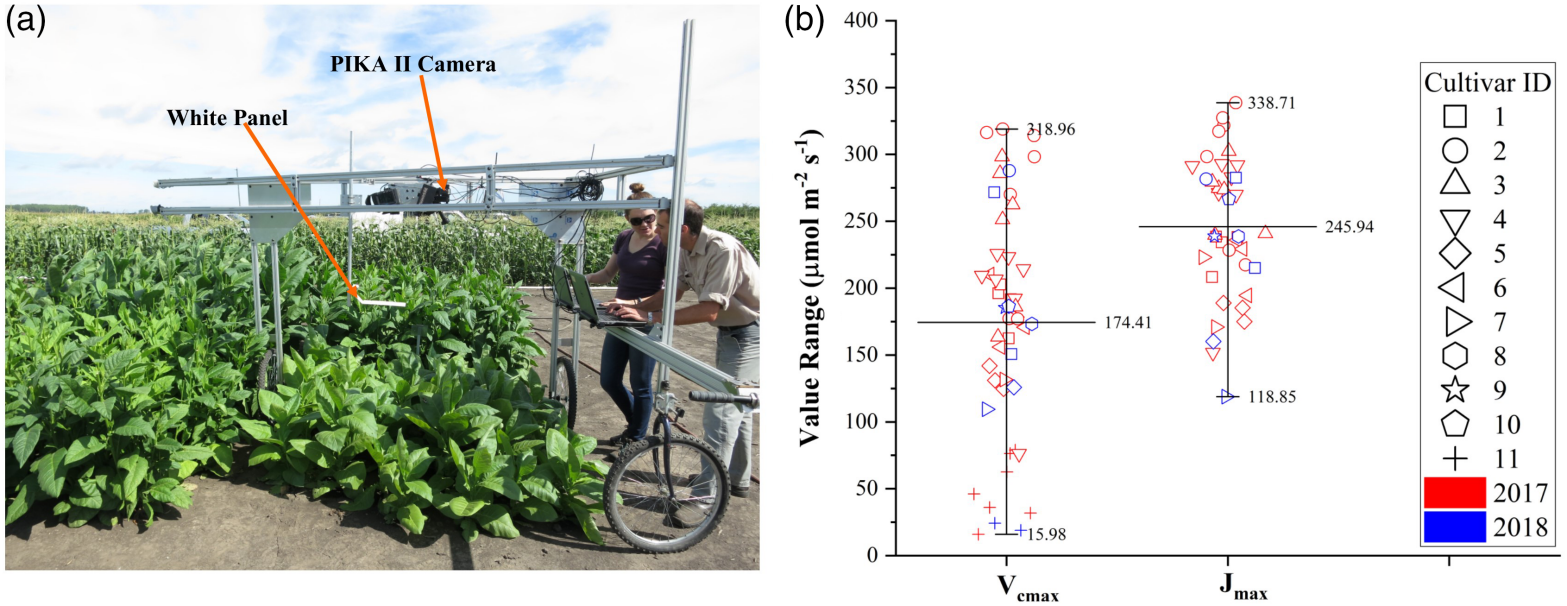
A ground‐based high‐throughput phenotyping platform (a) for collecting hyperspectral images and the value range for photosynthetic variables *V*_*cmax*_ and *J*_*max*_ (b). Measurements shown in panel (b) were made on June 22 and 26–27, July 6–7, 12, and 31, and August 18 in 2017 and July 24–25 in 2018 for 11 tobacco cultivars. The lines in (b) show the minimum, mean, and max values (from top to bottom) for *V*_*cmax*_ and *J*_*max*_

Eleven tobacco cultivars (referred to as 1–11) including both wild and genetically modified lines with large differences in photosynthetic traits (Figure 1) were used to assess performances of the three approaches to estimate photosynthetic traits. These genetically modified tobacco cultivars may show a *V*_*cmax*_ (*J*_*max*_) value larger than 300 μmol m^−2^ s^−1^, for example, due to increased carbon reduction enzymes (increase in the electron transport metabolite pools). Further details of these 11 cultivars can be found in Meacham‐Hensold et al. (2019).

These tobacco cultivars were first planted in greenhouse conditions for germination and then transplanted at the four‐leaf stage to the University of Illinois Energy Farm Facility (40.063° N, 88.207° W, further details about the farm are available at http://energyfarm.illinois.edu/index.html). Two weeks prior to transplanting, the field site was fertilized to 275 lbs./acre (~150 ppm) ESN Smart Nitrogen. Field experiments were conducted to collect hyperspectral images and leaf gas exchange during the 2017 and 2018 growing seasons (June–August). Cultivars were planted in replicated plots (*n* = 4), and each plant was arranged in a 6 × 6 grid (36 plants per plot) with 0.38‐m spacing between plantings. Irrigation was applied as needed to avoid water scarcity. To increase the representativeness of the collected datasets, leaf gas exchange and hyperspectral reflectance measurements (data pairs) were made at various dates (June 22 and 26–27, July 6–7, 12, and 31, and August 18 in 2017 and July 24–25 in 2018) to represent a range of phenological stages. In this study, a total of 48 data pairs were collected for *V*_*cmax*_ and 39 data pairs for *J*_*max*_ at the plot level. The fewer number of measurements for *V*_*cmax*_ than that for *J*_*max*_ resulted from one of the genetically modified tobacco cultivars not being electron transport limited under any conditions (removed from analysis as suggested in Fu et al. 2019).

### 2.2 Collection of hyperspectral imagery

A ground‐based high‐throughput phenotyping platform was fabricated to carry a series of sensors for collecting hyperspectral measurements (Figure 1). Because the focus of this study was mainly on tobacco plants in agricultural fields, the manually operated platform was adequate to provide spectral measurements over the whole study area within a few hours (completed within the time window between 11 a.m. and 2:30 p.m. local time). A Resonon PIKA II VNIR HSI camera was installed on the platform to collect hyperspectral images for each plot. The camera recorded spectral radiation from 400 to 900 nm in 2.1‐nm contiguous bands (240 spectral bands in total) with a push‐broom design. Images were collected at a height of 1.6 m from bare soil through a manual control system. Each scan consisted of 640 spatial channels along the row with a sampling distance of 0.1 mm (nadir view). A 99% reflective white panel (Spectralon, Labsphere Inc., North Dutton, NH, USA) was placed horizontally above canopy and was also scanned together with tobacco plants in the collected hyperspectral images. Exposure time was carefully set to avoid possible sensor saturation. For each plot, image acquisition and storage were completed in less than a minute.

### 2.3 Leaf gas exchange and chlorophyll measurements

Photosynthetic variables (*V*_*cmax*_ and *J*_*max*_) were derived from leaf gas exchange measurements provided by a portable infrared gas analyser (LI‐6400, LICOR Biosciences, Lincoln, NE, USA). This analyser recorded response of photosynthesis (A) to a series of intercellular CO_2_ concentration (*C*_*i*_) in a stepwise adjustment, that is, 400, 200, 50, 100, 300, 400, 600, 900, 1,200, 1,500, 1,800, and 2,000 (μmol mol^−^2). Within 30 min of image acquisition, gas exchange measurements were made on three sunlit, last fully expanded leaves per plot at saturating light of 1,800 μmol m^−2^ s^−1^ under clear‐sky conditions. Before initiating *A*/*C*_*i*_ curves, leaf temperature was measured using a FLIR TG54 handheld IR gun, and the block temperature was set to match this leaf temperature for the subsequent measurements. Before each curve measurement, relative humidity inside the chamber was manually controlled to 65% ± 5% by adjusting the flow through the desiccant tube integrated into the gas exchange system. Prior to the start of each curve, leaves were given a minimum of 200 s to adapt to chamber conditions. Measurements were made within 3 min at each CO_2_ step to minimize alteration of the activation state of Rubisco. Photosynthetic variables *V*_*cmax*_ and *J*_*max*_ were calculated by fitting a mathematical model (Bernacchi et al., 2004; Farquhar et al., 1980) with collected *A*/*C*_*i*_ curves (Sharkey et al., 2007). Mesophyll conductance was determined based on a previous study for tobacco at 25 ° (Evans & von Caemmerer, 2012). A total of 144 (117) leaf‐level samples (three leaf measurements from three plants per plot) were collected for *V*_*cmax*_ (*J*_*max*_). These *V*_*cmax*_ and *J*_*max*_ values were then respectively averaged over three leaves to provide estimates per plot, yielding 48 measurements for *V*_*cmax*_ and 37 measurements for *J*_*max*_ that were used for building predictive models.

To determine leaf chlorophyll content, tissues were extracted from leaves measured for gas exchange, immediately after gas exchange measurements, using a cork borer. Leaf disks (approximately 2.01 cm^2^) were placed in 2 mL tubes and flash frozen in liquid nitrogen. Each leaf disc was incubated in 96% (v/v) ethanol for 3 days at 4°C. The bleached material and ethanol were mixed (100 μL of solution for each sample) and analysed with synergy 2 photospectrometer (BioTek Instruments, Inc, Winooski, VT, USA) at 470, 649, and 665 nm. Chlorophyll *a* + *b* content was calculated according to Lichtenthaler and Wellburn (1983). The three measurements of chlorophyll *a + b* from different leaves were averaged to provide a single value per plot, which was compared with that derived from RTM‐based chlorophyll *a + b* for validation.

## 3 ANALYSIS TECHNIQUES

**Figure 2:**
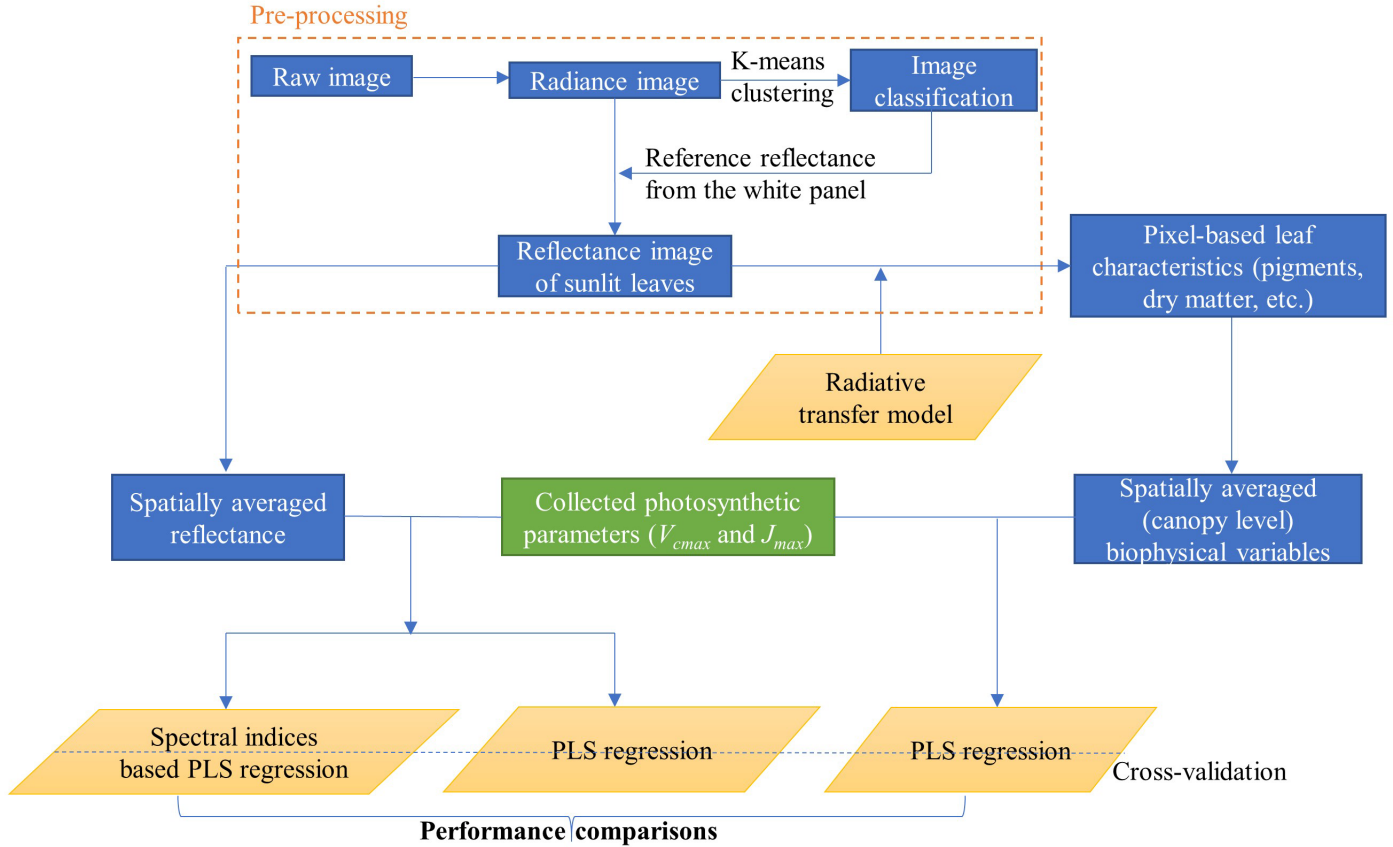
The overall workflow for data analysis in this study

Figure 2 shows the overall workflow for data analysis in this study. It consists of image‐preprocessing and further modelling of canopy‐level reflectance with photosynthetic variables *V*_*cmax*_ and *J*_*max*_ through PLSR and indices‐based analysis. An RTM designed specifically for close‐range remote sensing was applied to reflectance images of sunlit leaves to estimate biophysical variables on a per‐pixel basis. Spatially averaged biophysical parameters at plot level were correlated to *V*_*cmax*_ and *J*_*max*_ through PLSR. In this study, performance of each approach to predict *V*_*cmax*_ and *J*_*max*_ was assessed based on the coefficient of determination (*R* ^2^) and *RMSE*, which were calculated using a 10‐fold cross‐validation procedure due to a relatively small number of data pairs available. The cross validation was repeated for 1,000 times for predicting both *V*_*cmax*_ and *J*_*max*_.

### 3.1 Image preprocessing

**Figure 3:**
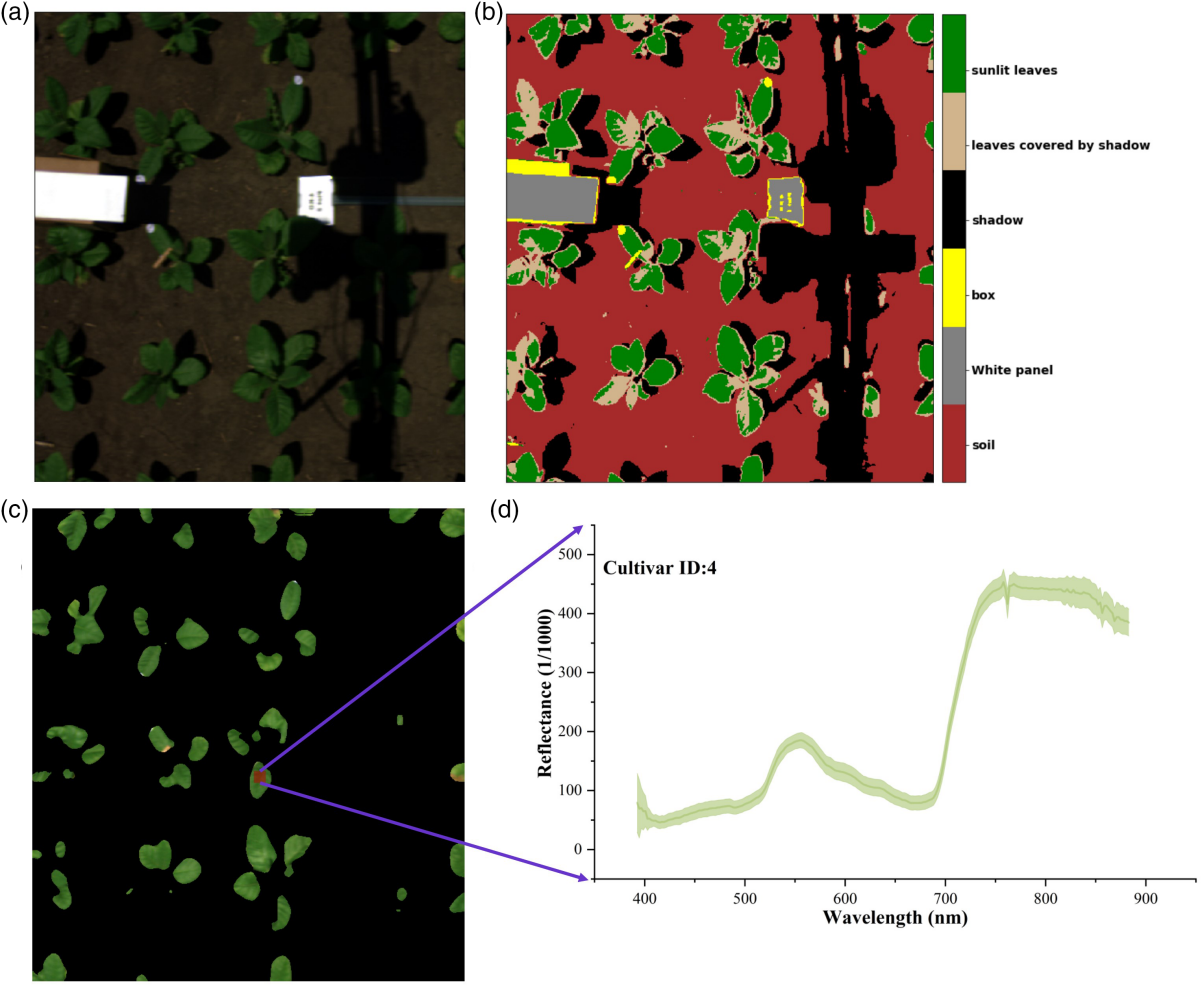
An example of data preprocessing steps to convert a raw image (a, RGB composite) to a reflectance image (c, RGB composite). Panel (b) is an image classified from (a) using the k‐means clustering algorithm, and panel (d) is a reflectance graph with shaded standard deviations for a region of interest (red rectangle) in (c)

Raw images captured by the HSI camera were stored in digital numbers with a 12‐bit depth. Figure 3a shows a raw image collected on June 27, 2017, for cultivar 4. The image preprocessing involved three phases: (1) radiometric calibration, (2) image classification, and (3) reflectance calculation. During radiometric calibration, digital numbers from raw data were converted to absolute spectral radiance (unit: W m^−2^ sr^−1^ μm^−1^) using calibration files provided by the instrument company. For image classification, an unsupervised classification algorithm, that is, k‐means clustering (Spath, 1985), was employed due to its simplicity. The number of clusters set in the algorithm was 6. To further identify the white panel and sunlit leaves from clusters, the following criteria were used. First, the cluster that had the highest mean radiance value was labelled as the white panel. Then both shadow‐covered and sunlit leaves were identified from clusters with a normalized difference VI value larger than 0.2. This threshold value worked for all the images collected in this study. Third, sunlit leaves were identified from one of the two clusters that exhibited a higher mean spectral radiance. Figure 3b presents an example of image classification using these steps, in which the reference panel and sunlit leaves were accurately identified. In the third phase of image preprocessing, reflectance was calculated using Equation (1).

(1) $$R=\frac{S_{\text{sunlit}}}{S_{\mathrm{ref}}}*R_{\mathrm{ref}}.$$

In Equation (1), *S*_*sunlit*_ and *S*_*ref*_ are radiance values from sunlit leaves and the white reference panel, respectively, *R*_*ref*_ refers to the reflectance of the white panel (calibrated) provided by Labsphere, and *R* is the absolute reflectance of sunlit leaves. These image preprocessing steps were coded in a python environment (Python Software Foundation, https://www.python.org/) to automatically convert collected raw images to reflectance images. This programming system is practically important in high‐throughput phenotyping of multiple crop cultivars at the plot level.

### 3.2 Partial least squares regression

The PLSR model was widely used to infer photosynthetic variables from hyperspectral reflectance at the leaf level (Dechant et al., 2017; Heckmann et al., 2017; Serbin et al., 2012; Yendrek et al., 2017). It is a bilinear regression technique that can reduce a large number of collinear spectral variables into several orthogonal components (also known as latent variables; Geladi & Kowalski, 1986; Wold et al., 2001). The model also projects the explained variables (*V*_*cmax*_ and *J*_*max*_ in this study) to a new space and then finds a linear regression model between the predicted variables and the independent variables in the new projection space (Geladi & Kowalski, 1986). Detailed descriptions of the PLSR algorithm can be found in (Ehsani et al., 1999). In general, PLSR can be formulated as Equations (2) and (3).

(2) $$y=\sum_{i=1}^{p}\alpha_{i}*{\mathrm{lv}}_{i},i=1,2,\ldots .p.$$

(3) $${\mathrm{lv}}_{i}=\sum_{j=1}^{m}\lambda_{j}*x_{j},i=1,2,\ldots .p.$$

where *y* is the predicted photosynthetic variable (*V*_*cmax*_ or *J*_*max*_), *p* is the number of latent variables used for regression, *α* refers to the regression coefficients, *lv* represents the latent component computed from the original input measurements *x* (with *m* as the dimension of input data), and λ is the eigenvector of *x* ^*T*^ *x* for the transformed latent component. Before PLSR, spectral data were normalized by computing the following metric: (raw‐mean)/std. The optimal number of latent variables was determined using the lowest RMSE of prediction from cross validations, following Esbensen et al. (2002), to prevent overfitting.

### 3.3 Spectral indices‐based analysis

Spectral indices based on two or three wavebands in the 400‐ to 900‐nm region were selected from the literature and examined to estimate photosynthetic variables among cultivars. Table 1 shows the three types of spectral indices used in this study, that is, simple ratio (*SR*; e.g., Clevers & Kooistra, 2012; Gitelson et al., 2003), modified normalized difference index (*mND*; e.g., Gitelson & Merzlyak, 1994), and structure insensitive pigment index (*SIPI*; e.g., Curran, 2004). These spectral indices were generally designed for estimating photosynthetic pigment contents (e.g., chlorophyll a) and structure characteristics at leaf and canopy levels and may have close associations with photosynthetic capacity (e.g., Croft et al., 2017). Unlike leaf pigment contents, photosynthetic variables *V*_*cmax*_ and *J*_*max*_ do not show obvious sensitivity to reflectance absorption and scattering in the spectral domain but are closely correlated with leaf characteristics (Serbin et al., 2012; Yendrek et al., 2017). The three types of spectral indices were mainly used in this study to extract spectral signatures from reflectance spectra within 400–900 nm.

**Table 1:** The type of spectral indices used in this study

Name of spectral index	Equation
Simple Ratio (SR)	$R_{\lambda_{1}}/R_{\lambda_{2}}$
Modified normalized difference index (mND)	$\left(R_{\lambda_{\mathrm{ref}}}-R_{\lambda_{1}}\right)/\left(R_{\lambda_{\mathrm{ref}}}+R_{\lambda_{2}}\right)$, *λ*_1_ ≠ *λ*_2_
structure insensitive pigment index (SIPI)	$\left(R_{\lambda_{\mathrm{ref}}}-R_{\lambda_{1}}\right)/\left(R_{\lambda_{\mathrm{ref}}}-R_{\lambda_{2}}\right)$

*Note*: *λ*_*ref*_ refers to the wavelength at 850 nm (near‐infrared band) or 440 nm (blue band) following Jay et al. (2017).

Because different band combinations can be used to compute these spectral indices, the optimal sets of wavebands were selected based on the correlation coefficient between spectral indices and photosynthetic variables. The performance of a spectral index to predict photosynthetic variables was evaluated using the squared Spearman's rank correlation coefficient (*ρ* ^2^). Compared with the Pearson's correlation coefficient (linear), the Spearman's coefficient emphasizes monotonic relationships (non‐linear or linear), which would be more suitable for mapping photosynthesis due to the possible non‐linear response of photosynthetic capacities to reflectance spectra. The band wavelength ranging from 400 to 900 nm was used to calculate the three types of spectral indices. In addition, for the *mND* and *SIPI* indices, *λ*_*ref*_ was set at 850 or 440 nm following Jay et al. (2017) to ensure that the indices conformed to their original forms. Spectral indices of each type that fell within the top 5% of correlation coefficient values (Hansen & Schjoerring, 2003) were selected as hyperspectral signatures. As this procedure may also lead to hundreds of spectral indices selected for analysis, the PLSR was further used to correlate these spectral indices with photosynthetic variables. The performance of the spectral indices‐based PLSR (including selection of spectral indices) was evaluated using *R* ^2^ and *RMSE*, derived from 1,000 cross validations.

### 3.4 Radiative transfer model

An RTM, called PROCOSINE and developed to describe and simulate leaf reflectance for close‐range imaging spectroscopy (Jay et al., 2016) on a per‐pixel basis, was used as the third approach to estimate photosynthetic variables. Based on the PROSPECT model version 5b (Feret et al., 2008), the newly developed close‐range RTM allows for direct and accurate estimations of foliar content from millimetre hyperspectral imagery using numerical inversions. As reported in Jay et al. (2016), the PROCOSINE model can retrieve leaf biochemical parameters with an *R* ^2^ of ~0.9 and an *RMSE* of less than 10% in laboratory conditions. Further technical details of the close‐range RTM can be found in Jay et al. (2016). The PROCOSINE model simulates leaf reflectance as a function of eight parameters on a per‐pixel basis: leaf structure parameter *N* (unitless), leaf chlorophyll *a + b* concentration *C*_*ab*_ (μg/cm^2^), carotenoid content *C*_*cx*_ (μg/cm^2^), brown pigment content *C*_*bp*_ (unitless), equivalent water thickness *C*_*w*_ (*cm*), leaf mass per area *C*_*m*_ (g/cm^2^), light incident angle *θ*_*i*_ (in °), and bidirectional reflectance distribution function effect *b*_*spec*_ (unitless). The PROCOSINE model can run in both backward (reflectance spectra > leaf traits) and forward (leaf traits > reflectance spectra) modes. The reduction of hyperspectral reflectance into eight parameters through the PROCOSINE model thus provides insights on what leaf characteristics are closely related with photosynthetic rates.

Inversion (or numerical solution) of the PROCOSINE model was achieved based on the widely used lookup table (LUT) approach (Dorigo, 2012; Richter et al., 2011). The LUT approach can help reduce computation load of non‐linear optimization of the eight parameters from the PROCOSINE model with collected reflectance spectra from 400 to 900 nm. To generate LUTs, the PROCOSINE model was run in the forward mode to simulate reflectance based on different sets of input variables. For each image, an LUT size of 100,000 entries was generated to balance computation load and model inversion accuracy (Marie et al., 2000). Input variables in each entry were randomly generated from the specific ranges as shown in Table 2 with uniform distributions (Equation 4).

(4) $$V_{i}=V_{\min}+\left(V_{\max}-V_{\min}\right)*{\mathrm{rand}}_{i},i=1,\ldots 8.$$

where *V*_*i*_ is the variable value, *V*_*min*_ and *V*_*max*_ are the minimum and maximum value of the *i* ^*th*^ variable (Table 2), and *rand*_*i*_ is a uniformly distributed random number within 0–1.

**Table 2:** Ranges of each input variable to generate lookup tables for inverting the PROCOSINE model

Variable	Unit	Minimum	Maximum
Leaf structure parameter *N*	Unitless	1	3
Leaf chlorophyll *a + b* concentration *C*_*ab*_	μg/cm^2^	0	100
Carotenoid content *C*_*cx*_	μg/cm^2^	0	30
Brown pigment content *C*_*bp*_ a	Unitless	0	0
Equivalent water thickness *C*_*w*_	cm	0.0005	0.1
Leaf mass per area *C*_*m*_	g/cm^2^	0.001	0.1
Light incident angle *θ*_*i*_	°	0	90
BRDF effect *b*_*spec*_	Unitless	−0.2	0.6

a Brown pigment content was set to zero because senescent leaves were not observed in the field experiments.

Abbreviation: BRDF, bidirectional reflectance distribution function.

To further constrain the model, brown pigment content was set to zero because senescent leaves were not observed in the fields over the study period. After the LUT generation, the *RMSE* between the measured and simulated leaf reflectance was calculated for each LUT entry on a per‐pixel basis. The LUT entries of the 10 smallest *RMSE* values were averaged as the solution to the inverse problem to reduce uncertainty (Darvishzadeh et al., 2011). The seven parameters on a per‐pixel basis were then individually averaged to plot‐level variables, which were further correlated with photosynthetic variables using the PLSR. The performance of the RTM‐based PLSR was assessed using *R* ^2^ and *RMSE*, derived from 1,000 cross validations.

### 3.5 Spectral resampling

Spectral resampling generally involves convolving reflectance spectra to wider wavelength intervals around selected band centres or to the spectral configuration of existing sensors (Adjorlolo et al., 2013). In this study, the original reflectance spectra were resampled at different spectral resolutions to investigate the impacts of spectral regions on the PLSR performance for predicting photosynthetic capacities. The objective of this analysis is to provide insights about whether a hyperspectral camera can be replaced by a multispectral camera to quantify photosynthetic traits in a high‐throughput manner.

**Figure 4:**
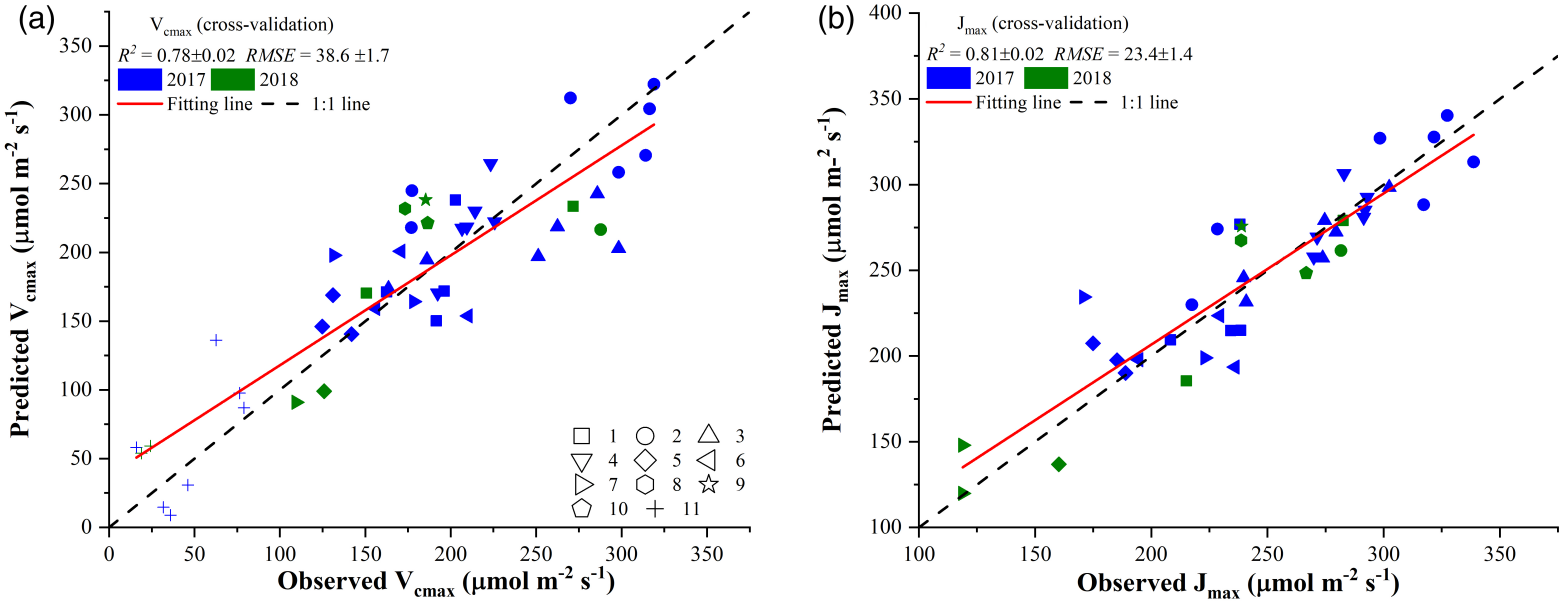
Comparisons between observed photosynthetic variables, *V*_*cmax*_ (a) and *J*_*max*_ (b), and those predicted from plot‐level hyperspectral reflectance imaging using the partial least square regression. The value of each predicted point in the scatter plot was the mean value of 1,000 cross‐validation predictions. The standard deviations of *R* ^2^ and *RMSE* were also provided based on the 1,000 cross validations. The shape of scatter points refers to the type of tobacco cultivars

As the HSI camera used in this study has 240 spectral bands, it is impossible to examine the impacts of different band combinations (after convolving the original reflectance spectra to wider band intervals by selecting each of them as the band centre) on the predictive performance of PLSR (240 permutations for band combinations). Thus, important band centres were selected for the convolution of the original reflectance spectra at different bandwidth intervals (4.2, 6.3, …, and 21 nm) from 400 to 900 nm. Important band centres were chosen based on a threshold set on the absolute value of the PLS regression coefficient at each wavelength (these coefficients were used to predict *V*_*cmax*_ and *J*_*max*_ as shown in Figure 4). The threshold values used to select important spectral band centres ranged from 0 (all spectral bands were used) to 0.6 (only five to seven bands were used) with an interval of 0.05. For each band centre, a Gaussian model with a full width at half maximum (FWHM; Adjorlolo et al., 2013) equivalent to the specified bandwidth interval (4.2, 6.3, …, and 21 nm) was utilized for convolution of the original reflectance spectra (as shown in Equation 5). After each spectral resampling, the performance of the PLSR (*R* ^2^ and *RMSE*) with inputs of new reflectance spectra (1,000 cross validations) to predict photosynthetic parameters *V*_*cmax*_ and *J*_*max*_ was recorded for comparisons.

(5) $$\left\{\begin{array}{c} R_{\mathrm{co}nv}=\frac{\int_{\lambda_{1}}^{\lambda_{2}}R_{0}W_{\lambda }d\lambda }{\int_{\lambda_{1}}^{\lambda_{2}}W_{\lambda }d\lambda }\\ W_{\lambda }=\frac{1}{\delta \sqrt{2\pi }}\exp\left[-\frac{{\left(\lambda -\lambda_{0}\right)}^{2}}{2\delta^{2}}\right]\\ \text{FWHM}=2\sqrt{2\ln2}\delta \end{array}\right..$$

where *R*_*conv*_ refers to resampled reflectance spectra, *R*_0_ refers to original reflectance spectra, *W*_*λ*_ is the weight per wavelength derived from the Gaussian model with the FWHM equivalent to the specified bandwidth interval, *δ* is the standard deviation related to FWHM, and λ_0_ is the band centre selected using the threshold set to the absolute value of the PLS regression coefficients.

## 4 RESULTS

### 4.1 The predictive performance of PLSR with reflectance spectra

Figure 4 presents comparisons between the observed photosynthetic variables (*V*_*cmax*_ and *J*_*max*_) and those estimated from plot‐level reflectance spectra using the PLSR. The cross‐validation results showed that *V*_*cmax*_ and *J*_*max*_ could be well predicted from reflectance spectra with an *R* ^2^ of 0.78 ± 0.02 and 0.81 ± 0.02, respectively, and with an *RMSE* of 38.6 ± 1.7 and 23.4 ± 1.4 μmol m^−2^ s^−1^, respectively, at the plot level. For *V*_*cmax*_ predictions, it was observed that the PLSR model tended to underestimate values that were greater than 250 μmol m^−2^ s^−1^. However, the prediction uncertainty over the 1,000 cross‐validation predictions was quite small as evidenced by the small standard deviations in *R* ^2^ (0.02) and *RMSE* (1.7). Compared with the *R* ^2^ for *V*_*cmax*_, a larger *R* ^2^ value for *J*_*max*_ was found (Figure 4b).

### 4.2 The predictive performance of PLSR with hyperspectral signatures

**Figure 5:**
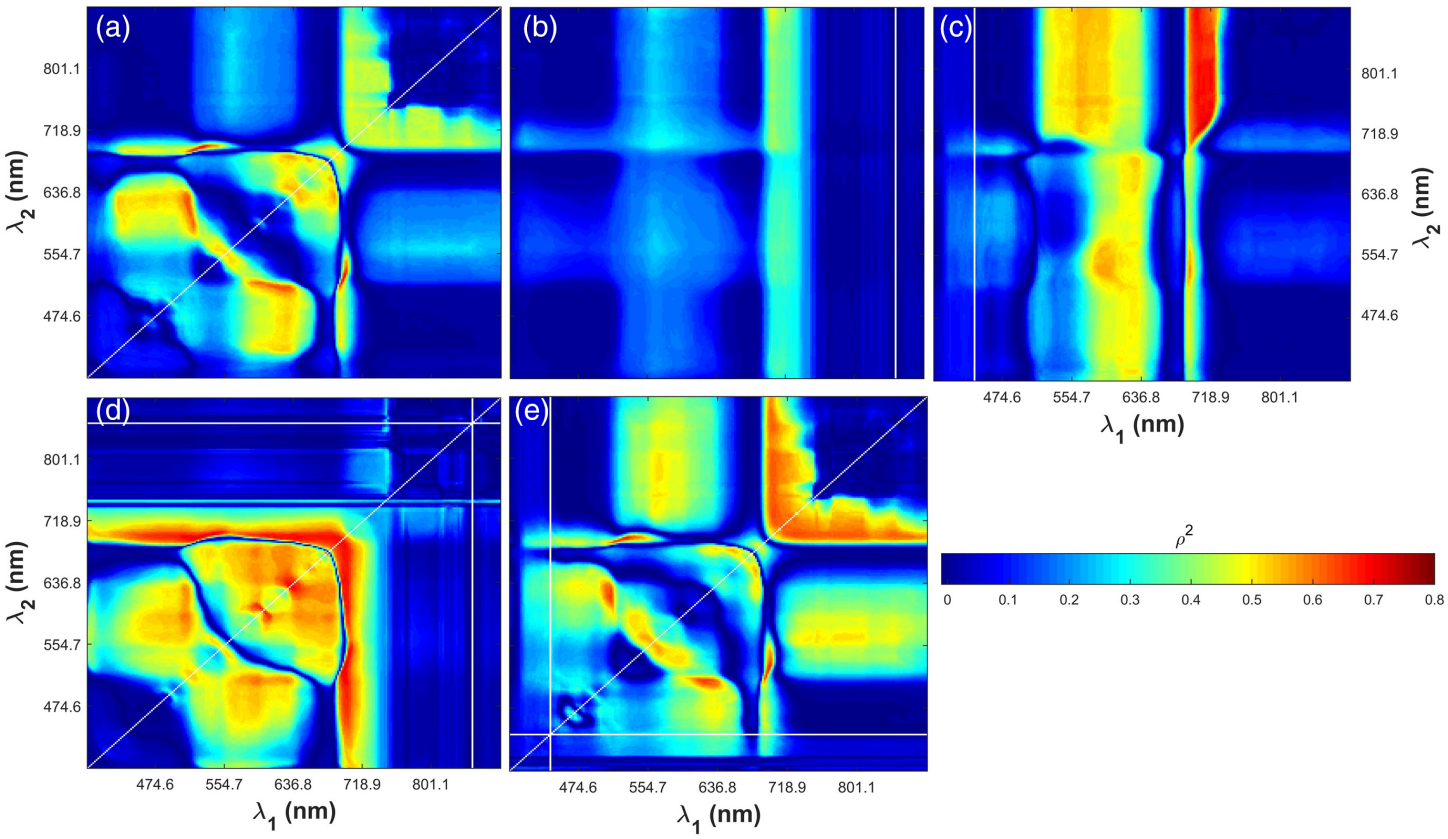
The squared correlation coefficients (*ρ* ^2^) between *V*_*cmax*_ and spectral indices in the form of simple ratio (a), modified normalized difference index with the reference band at 850 nm (b) and 440 nm (c), and structure insensitive pigment index with the reference band at 850 nm (d) and 440 nm (e). The equations of these spectral indices were $R_{\lambda_{1}}/R_{\lambda_{2}}$ for *SR*, $\left(R_{\lambda_{\mathrm{ref}}}-R_{\lambda_{1}}\right)/\left(R_{\lambda_{\mathrm{ref}}}+R_{\lambda_{2}}\right)$, *λ*_1_ ≠ *λ*_2_ for *mND*, and $\left(R_{\lambda_{\mathrm{ref}}}-R_{\lambda_{1}}\right)/\left(R_{\lambda_{\mathrm{ref}}}-R_{\lambda_{2}}\right)$ for *SIPI*. *λ*_*ref*_ refers to the wavelength at 850 nm (near‐infrared band) or 440 nm (blue band)

**Figure 6:**
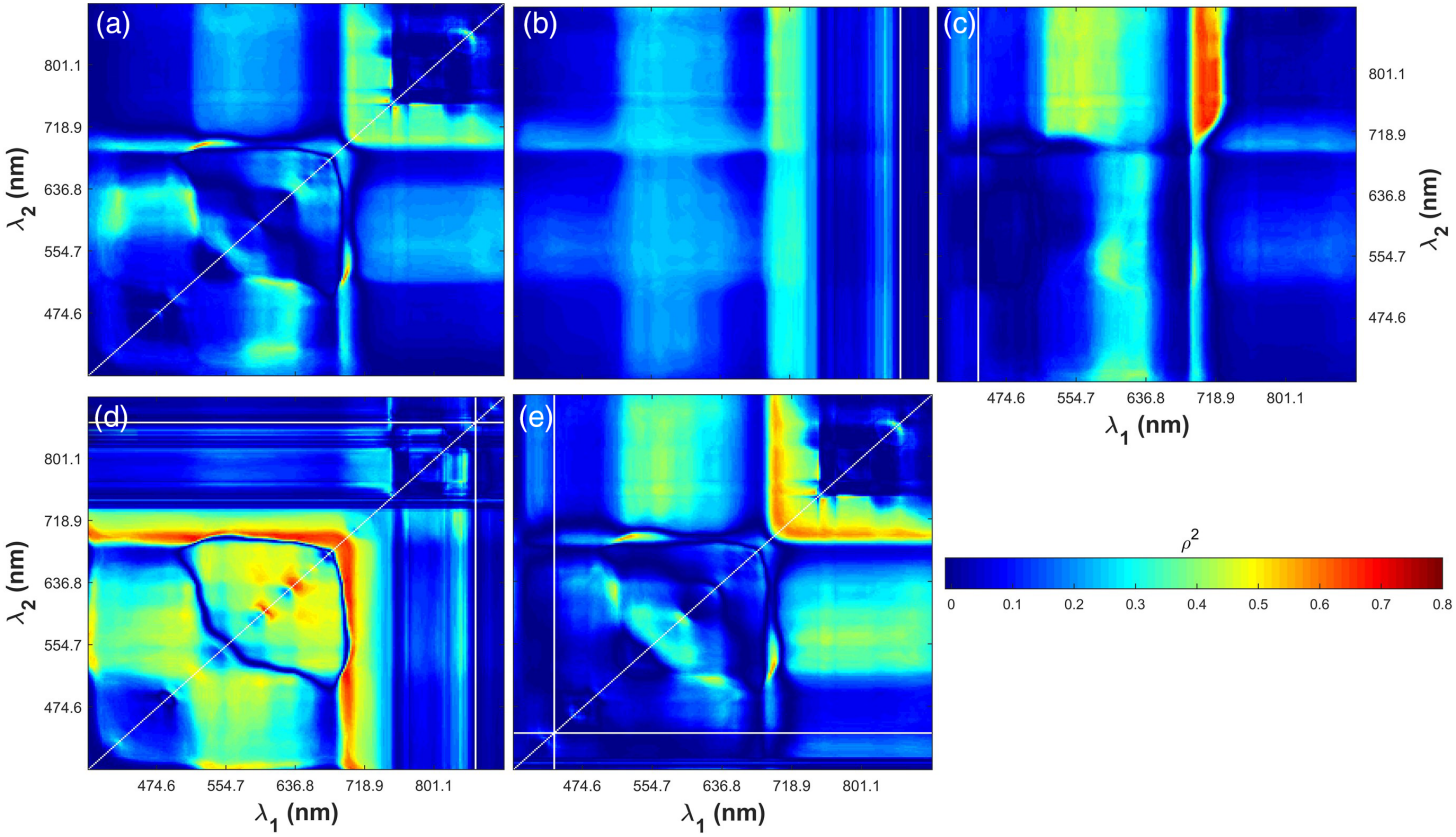
The squared correlation coefficients (*ρ* ^2^) between *J*_*max*_ and spectral indices in the form of simple ratio (a), modified normalized difference index with the reference band at 850 nm (b) and 440 nm (c), and structure insensitive pigment index with the reference band at 850 nm (d) and 440 nm (e). The equations of these spectral indices were $R_{\lambda_{1}}/R_{\lambda_{2}}$ for *SR*, $\left(R_{\lambda_{\mathrm{ref}}}-R_{\lambda_{1}}\right)/\left(R_{\lambda_{\mathrm{ref}}}+R_{\lambda_{2}}\right)$, *λ*_1_ ≠ *λ*_2_ for *mND*, and $\left(R_{\lambda_{\mathrm{ref}}}-R_{\lambda_{1}}\right)/\left(R_{\lambda_{\mathrm{ref}}}-R_{\lambda_{2}}\right)$ for *SIPI*. *λ*_*ref*_ refers to the wavelength at 850 nm (near‐infrared band) or 440 nm (blue band)

Figures 5 and 6 show the squared correlation coefficients (*ρ* ^2^) and spectral indices for *V*_*cmax*_ and *J*_*max*_, respectively. These spectral indices were in the form of *SR* (a), *mND* with the reference band at 850 nm (b) and 440 nm (c), and *SIPI* with the reference band at 850 nm (d) and 440 nm (e). Hotspots with high correlation coefficients (red colours in Figures 5 and 6) were identified for all possible band combinations of the reflectance measured at 240 wavelengths. For example, Figures 5c and 6c show that *V*_*cmax*_ and *J*_*max*_ can be predicted with a *ρ* ^2^ larger than 0.7 using *mND* (reference band at 440 nm), that is, *λ*_1_ between 700 and 720 nm and *λ*_2_ between 710 and 810 nm. The high correlation of this identified spectral region with total chlorophyll and nitrogen contents, leaf mass, and LAI (Carter, 1994; Curran, 1994; Horler et al., 1983) may explain the observed hotspots in Figures 5c and 6c. In addition, Figures 5a,d,e and 6a,d,e also highlight important spectral regions from 470 to 530 nm and from 560 to 660 nm, respectively. These spectral regions were strongly associated with leaf pigment and nitrogen contents as well as light absorption of chlorophyll *a* (Blackburn, 1998; Carter, 1994; Faurtyot & Baret, 1997; Gamon et al., 1992). Among all the spectral indices, *mND* with the reference band at 850 nm (Figures 5b and 6b) overall exhibited the worst performance (squared correlation coefficients were well below 0.6) for predicting both *V*_*cmax*_ and *J*_*max*_. Compared with the squared correlation coefficients in Figure 5 (for *V*_*cmax*_), those in Figure 6 (for *J*_*max*_) were relatively smaller, which probably could be explained by the narrow value range of *J*_*max*_ (Figure 1b) due to a limited number of ground‐truth samples.

**Figure 7:**
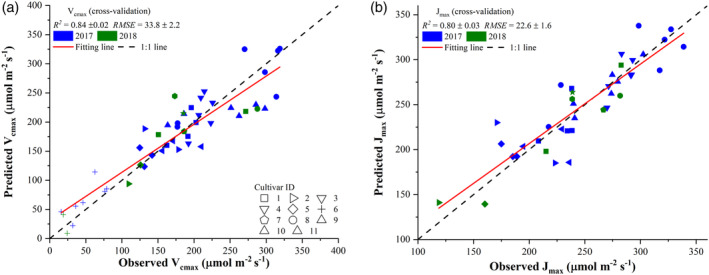
Comparisons between observed photosynthetic variables, *V*_*cmax*_ (a) and *J*_*max*_ (b), and those predicted from plot‐level hyperspectral signatures (spectral indices) using the partial least square regression. The value of each predicted point in the scatter plot was the mean value of 1,000 cross‐validation predictions. The standard deviations of *R* ^2^ and *RMSE* were also provided based on the 1,000 cross validations. The shape of scatter points refers to the type of tobacco cultivars

Figure 7 presents comparisons between observed photosynthetic variables, *V*_*cmax*_ (a) and *J*_*max*_ (b), and those predicted from plot‐level hyperspectral signatures (spectral indices) using the PLSR. The value of each predicted point in the scatter plot was the mean value of 1,000 cross‐validation predictions. Only spectral indices of each category with the correlation coefficient value falling within the top 5% were used to highlight the contributions of important spectral regions to the prediction performance. The selection of top 5% correlation coefficient values led to a total of 617 spectral indices used for predicting *V*_*cmax*_ and a total of 243 spectral indices used for predicting *J*_*max*_. With these selected hyperspectral signatures and PLSR, *V*_*cmax*_ can be predicted with an *R* ^2^ of 0.84 ± 0.02 and *RMSE* of 33.8 ± 2.2 μmol m^−2^ s^−1^ (Figure 7a). More specifically, the use of hyperspectral signatures improved the modelling performance by an increase of 0.06 (7.7%) in *R* ^2^ and a reduction of 4.8 (12.4%) in *RMSE* compared with those in Figure 4. With the selected spectral indices, the PLSR yielded an *R* ^2^ of 0.80 ± 0.03 and *RMSE* of 22.6 ± 1.6 μmol m^−2^ s^−1^ for predicting *J*_*max*_ at plot level. Although the use of spectral indices did not improve the *R* ^2^ value, it led to a reduction of *RMSE* by 0.8 (3.4%).

### 4.3 The predictive performance of PLSR with RTM‐based variables

**Figure 8:**
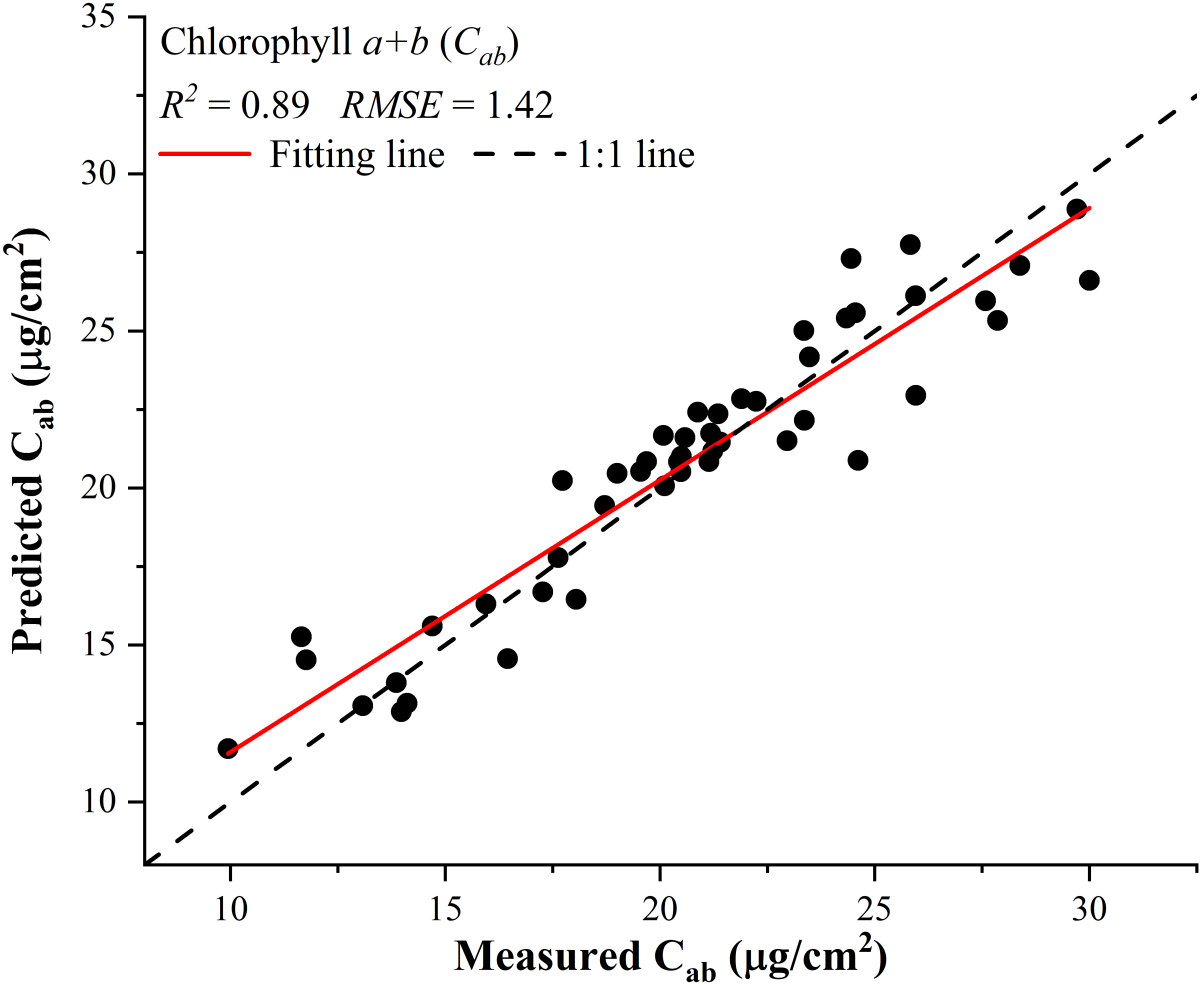
Comparisons between measured chlorophyll *a + b* (*C*_*ab*_) and that predicted from the radiative transfer model PROCOSINE

**Figure 9:**
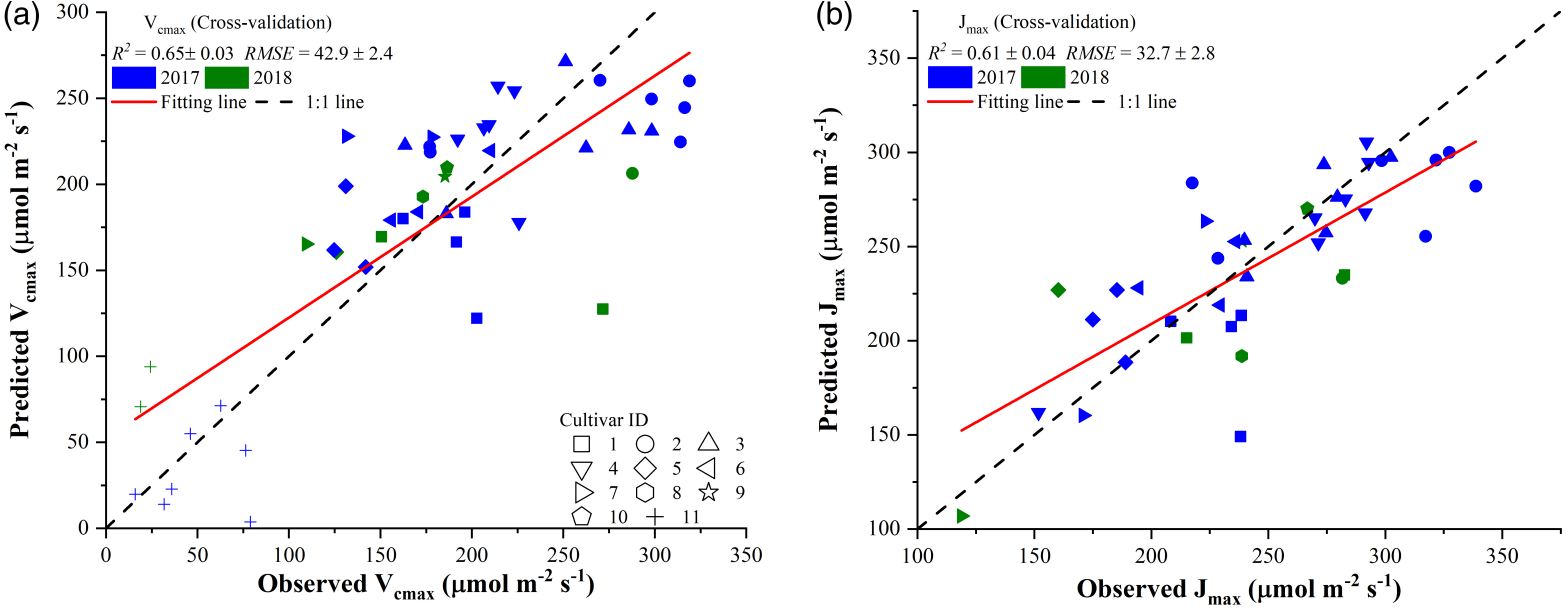
Comparisons between observed photosynthetic variables, *V*_*cmax*_ (a) and *J*_*max*_ (b), and those predicted from radiative transfer model‐derived variables using the partial least square regression. The value of each predicted point in the scatter plot was the mean value of 1,000 cross‐validation predictions. The standard deviations of *R* ^2^ and *RMSE* were also provided based on the 1,000 cross validations. The shape of scatter points refers to the type of tobacco cultivars

Using the LUT approach, the inversion of RTM PROCOSINE on a per‐pixel basis overall exhibited an *R* ^2^ of 0.82 ± 0.03 and an *RMSE* of 0.022 ± 0.01 for comparing the simulated and measured reflectance spectra from 400 to 900 nm. The performance of the PROCOSINE model was also evaluated by the comparisons between measured and predicted Chlorophyll *a + b* concentration with an *R* ^2^ of 0.89 and an *RMSE* of 1.42 μg/cm^2^ (Figure 8). Based on the RTM‐derived parameters, that is, leaf structure parameter *N*, leaf chlorophyll *a + b* concentration *C*_*ab*_, carotenoid content *C*_*cx*_, equivalent water thickness *C*_*w*_, leaf mass per area *C*_*m*_, light incident angle *θ*_*i*_, and bidirectional reflectance distribution function effect *b*_*spec*_, the PLSR yielded an *R* ^2^ of 0.65 ± 0.03 with an *RMSE* of 42.9 ± 2.4 μmol m^−2^ s^−1^ for predicting *V*_*cmax*_ (Figure 9a) and an *R* ^2^ of 0.61 ± 0.04 with an *RMSE* of 32.7 ± 2.8 μmol m^−2^ s^−1^ for predicting *J*_*max*_ (Figure 9b).

### 4.4 The impacts of spectral resolution on PLSR performance

**Figure 10:**
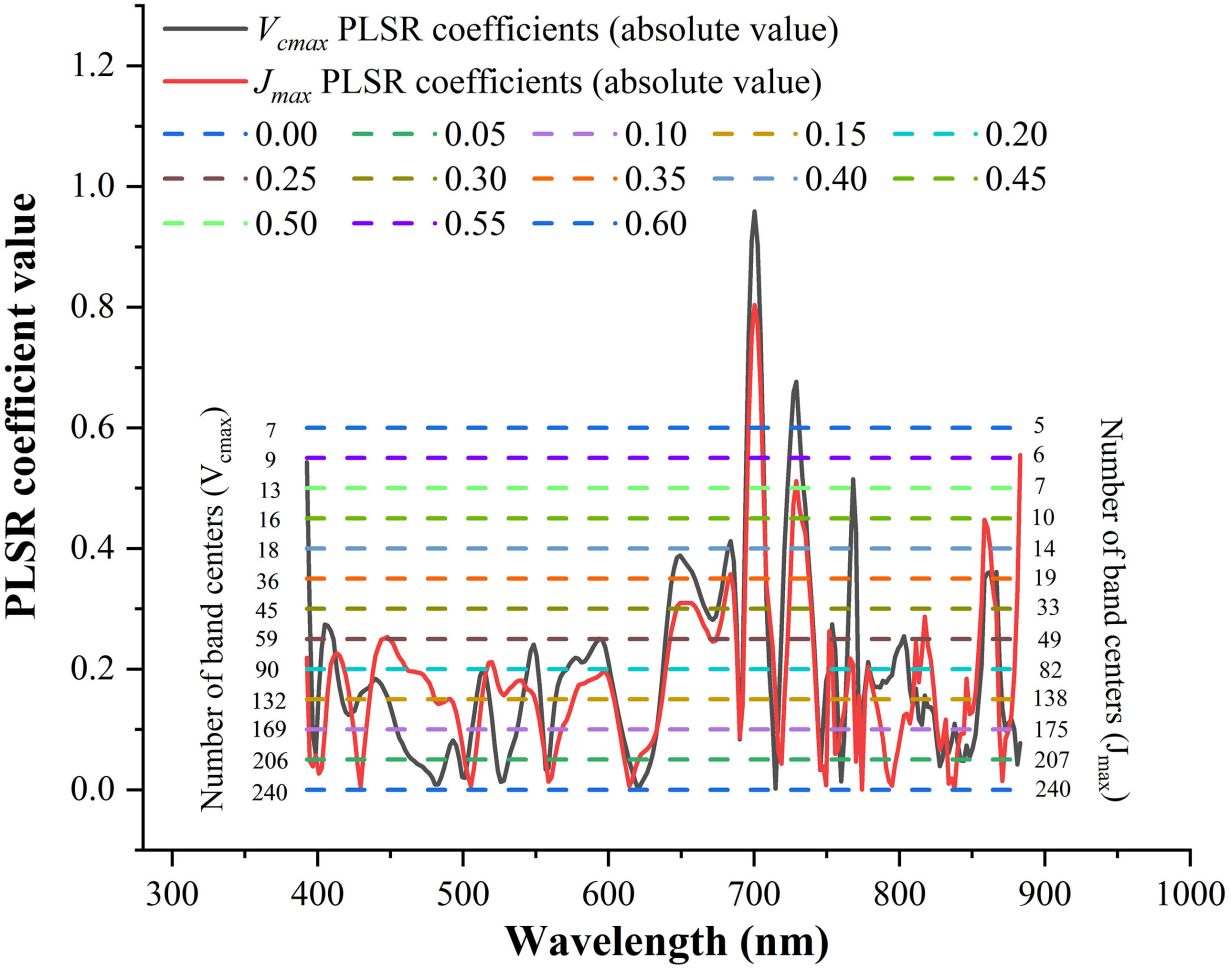
The partial least square regression coefficients (absolute value) used for predicting *V*_*cmax*_ and *J*_*max*_ (solid lines), the threshold (value ranged from 0 to 0.6) used to select band centres (dash lines), and the number of band centres used for convolution at each threshold

The impacts of spectral resolution on *V*_*cmax*_ and *J*_*max*_ predictions were examined through PLSR with resampled reflectance spectra as inputs rather than with spectral indices and RTM‐based traits as inputs. The reason was that band centres were hard to identify in the RTM‐based approach and that band centres were similar between reflectance and spectral indices‐based approaches (as suggested by Figures 5, 6, and 10). Figure 10 shows the absolute value of PLSR coefficients used for predicting *V*_*cmax*_ and *J*_*max*_ (cross‐validation predictions shown in Figure 4 were based on these coefficients). These absolute coefficient values highlighted important spectral regions such as wavelengths around 660, 700, and 720 nm, and similar important spectral regions were also identified using the spectral indices‐based correlation analysis (Figures 5 and 6). These important spectral regions were also consistent with previous studies such as Serbin et al. (2012) and Yendrek et al. (2017). With the threshold value ranging from 0 to 0.6 (with an interval of 0.05), the number of band centres used for spectral convolution varied from 240 to 7 for *V*_*cmax*_ and from 240 to 5 for *J*_*max*_ (Figure 10).

**Figure 11:**
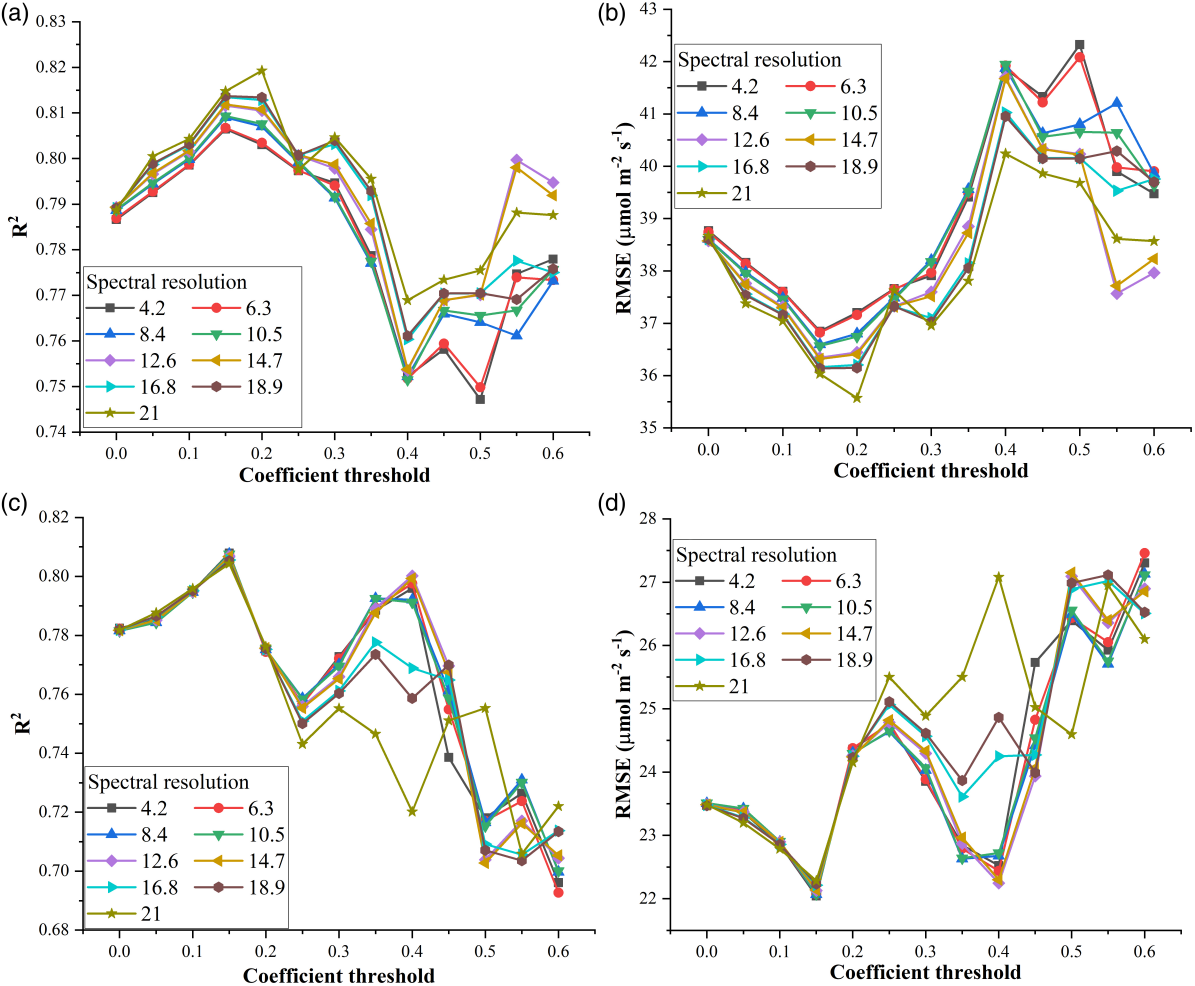
The predictive performance (*R* ^2^ and *RMSE*) of partial least square regression (PLSR) with inputs of reflectance spectra for predicting *V*_*cmax*_ (a,b) and *J*_*max*_ (c,d) at different spectral resolutions (4.2, 6.3, …, and 21.0 nm) and different coefficient threshold. Band centres were selected based on a series of PLSR band‐specific coefficient thresholds (value ranged from 0 to 0.6) set on the absolute values of PLSR regression coefficients (These regression coefficients were used for cross validations as shown in Figure 4)

Figure 11 presents the performance (*R* ^2^ and *RMSE*) of PLSR with resampled reflectance spectra as inputs for predicting *V*_*cmax*_ (a,b) and *J*_*max*_ (c,d) at each threshold with different spectral resolutions. Generally, as the threshold value varied from 0 to 0.6, two peaks in *R* ^2^ at the threshold coefficients of 0.15 and 0.55 emerged for predicting *V*_*cmax*_ (Figure 11a). More specifically, an increased trend in *R* ^2^ was observed as the coefficient threshold value ranged from 0 to 0.15 and from 0.4 to 0.6, and a decreased trend in *R* ^2^ was found as the coefficient threshold value changed from 0.15 to 0.4. The corresponding *RMSE* value varied in an opposite direction as shown in Figure 11b. These findings suggested that there existed an optimal number of spectral bands (132) used for predicting *V*_*cmax*_ and more than or less than this number of spectral bands could reduce the predictive performance of PLSR. Even though there was a peak in *R* ^2^ as threshold coefficient varied from 0.4 to 0.6, the peak *R* ^2^ values were generally less than those observed at the threshold coefficient of 0.15. In addition, it was found that *R* ^2^ values across different spectral resolution at the threshold coefficient value of 0.6 were very similar to those at the threshold coefficient value of 0 (with difference in *R* ^2^ no more than 0.04 between the two thresholds). These similar *R* ^2^ values indicated that it may be possible to predict *V*_*cmax*_ using a multispectral camera (with seven spectral bands at the threshold value of 0.6). However, such a conclusion requires further modelling efforts with a customized camera with spectral regions around 700 and 720 nm (Figure 10). As spectral resolution increased, the *R* ^2^ (*RMSE*) generally displayed an increase (decrease) trend for predicting *V*_*cmax*_ with more fluctuations at the threshold values of 0.55 and 0.6, which may highlight impacts from both the number of band centres and spectral resolutions on the predictive performance of PLSR.

For *J*_*max*_ predictions, *R* ^2^ across different coefficient threshold values exhibited three major peaks at 0.15, 0.4, and 0.55, respectively (Figure 11c). The highest peak *R* ^2^ value was 0.81, similar to that in Figure 4. As the coefficient threshold value varied from 0 to 0.2, spectral resolution only exerted minor impacts on the *R* ^2^ and *RMSE* values, evidenced by similar *R* ^2^ and *RMSE* values across different spectral resolutions within this threshold range (Figure 11c,d). In contrast, large fluctuations (high standard deviations) in *R* ^2^ and *RMSE* across different spectral resolutions were observed with the coefficient threshold value ranging from 0.2 to 0.6 (particularly at the coefficient threshold value of 0.4). This phenomenon may suggest the number of band centres rather than spectral resolution as a major factor affecting the performance of PLSR for predicting *J*_*max*_. At the coefficient threshold value of 0.4, the highest *R* ^2^ value was 0.80 with the spectral resolution set at 6.3 nm. This *R* ^2^ value was consistent with the peak value at the coefficient threshold value of 0.15 and that provided in Figure 4. This finding suggested that *J*_*max*_ could be predicted using a reduced number of spectral bands with a same modelling performance as predictions using all 240 spectral bands. As indicated in Figure 10, the number of band centres was 14 with the coefficient threshold value set as 0.4. The removal of spectral redundancy and collinearity may explain a still good performance of PLSR, that is, the reduction of spectral bands from 240 to 14 may largely remove spectral redundancy and collinearity that exist among all the 240 spectral bands. These results also indicated that it may be possible to predict *J*_*max*_ using a multispectral camera with spectral regions around 700 and 720 nm but with a spectral resolution smaller than 16.8 nm.

## 5 DISCUSSION

### 5.1 Mapping photosynthetic traits at the canopy level

This study provided direct evidence that mapping *V*_*cmax*_ and *J*_*max*_ at the canopy level could be successful with reflectance spectra (or derived variables) from 400 to 900 nm used as predictors. The findings shown in Figure 4 were in agreement with previous studies to show the PLSR model as an effective tool to predict photosynthetic variables across different spatial scales (Ainsworth et al., 2014; Dechant et al., 2017; Fu et al., 2019; Meacham‐Hensold et al., 2019; Serbin et al., 2012; Serbin et al., 2015). However, this study only used reflectance spectra from 400 to 900 nm for the PLSR modelling, which exhibited an even higher or at least similar *R* ^2^ value compared with previous modelling results at the leaf level (e.g., Dechant et al., 2017). Results presented in this study showed that *V*_*cmax*_ and *J*_*max*_ could be well estimated for 11 cultivars of one crop (including both genetically modified and wild types) using the proposed three approaches (i.e., PLSR of reflectance spectra, spectral indices, and RTM‐inversion of crop traits) with *R* ^2^ all larger than 0.6. These modelling performances at the canopy level using reflectance spectra from 400 to 900 nm were even better than those at the leaf level using a similar dataset from the whole spectrum (400–2,500 nm; Fu et al., 2019; Meacham‐Hensold et al., 2019). A similar finding was also observed in comparisons between Serbin et al. (2012) and Serbin et al. (2015) for tree species: *V*_*cmax*_ were better predicted at the canopy level (*R* ^2^ = 0.94) than at the leaf level (*R* ^2^ = 0.89). These results may indicate that the spatial averaging of photosynthetic parameters and pixel‐based reflectance spectra removed intraplot variations that can be seen from leaf‐level analysis and potentially led to better predictive performances at the plot level. Another possible reason for the better predictions at the canopy level than the leaf level may be associated with plant geometry structure. For example, leaves from plants with higher *V*_*cmax*_ and *J*_*max*_ tend to be flatter and thus can provide more homogeneous reflectance measurements that would improve the modelling performance. However, further research is needed to test this hypothesis.

Compared with Serbin et al. (2015), predictive performance of *V*_*cmax*_ (*R* ^2^ = 0.78) presented in this study was relatively worse. In our study, *V*_*cmax*_ was determined at field temperatures rather than normalized to a reference temperature as done previously (Serbin et al., 2015). *V*_*cmax*_ has a strong temperature dependence typical of enzymatically driven reactions (e.g., Bernacchi et al., 2001); thus, variation in temperature does not reflect changes in Rubisco content (Dechant et al., 2017) suggesting that the temperature response may not be detected using reflectance spectra. In addition, the relatively worse performance of *V*_*cmax*_ predictions in this study was partly attributed to the use of reflectance spectra from 400 to 900 nm rather than the full spectrum (excluding water absorption bands) in Serbin et al. (2015). However, it is expected that the modelling performance from the full spectrum for predicting *V*_*cmax*_ and *J*_*max*_ should be very similar to that from the spectral regions (400–900 nm) used in this study, evidenced by results at the leaf level in Fu et al., (2019) and Dechant et al. (2017).

In this study, a better performance of the PLSR approach with spectral indices as inputs, compared with that with reflectance spectra as inputs, for predicting *V*_*cmax*_ (higher *R* ^2^ and lower *RMSE* values) and *J*_*max*_ (similar *R* ^2^ but lower *RMSE* values) was observed. This should be attributed to the fact that the optimized spectral indices were generally less sensitive to the errors in absolute reflectance measurements. As the spectra‐based PLSR approach critically depended on the accuracy of absolute reflectance spectra, subtle changes in sky (e.g., directional illumination) and weather conditions (e.g., wind speed), which can introduce variations in measured reflectance spectra, may lead to uncertainties in predictions of *V*_*cmax*_ and *J*_*max*_. However, because these spectral indices were optimized to predict photosynthetic traits for tobacco plants, the spectral indices‐based PLSR approach may be less generalizable (i.e., a relatively worse prediction performance) to extend to other plant species than spectral‐based PLSR approach (e.g., Yang et al., 2016).

*V*_*cmax*_ and *J*_*max*_ predictions with an *R* ^2^ around 0.8 using reflectance‐based and spectral indices‐based approaches (Figures 4 and 7) further suggested that plot‐level high‐throughput phenotyping of photosynthetic traits could be successful. This result is of importance to further advance and speed up plant breeding processes using hyperspectral sensors onboard different sensing platforms (e.g., gantries, robotics, and unmanned aerial vehicles) that can easily scan a relatively larger number of crop cultivars in an automatic manner. The automatic collection of hyperspectral images with close‐range or remote sensing platforms would relieve the efforts to collect reflectance spectra using hand‐held devices such as FieldSpec4 (Analytical Spectral Devices, Boulder, Colorado). Plus, the use of reflectance spectra from 400–900 nm only and the potential replacement of a hyperspectral camera by a multispectral camera (as shown in Figure 11) can lower the payloads on sensing platforms (particularly useful for unmanned aerial vehicles‐based sensing platforms) as well as phenotyping costs. However, as the variance in *V*_*cmax*_ and *J*_*max*_ (as shown in Figure 1b) for tobacco cultivars is generally larger than that for food crops such as maize and soybean, research efforts are still needed to evaluate the developed approaches in high‐throughput phenotyping of food crops for desired photosynthetic improvements. More importantly, there is a need to explore whether the developed approaches for estimating photosynthetic capacities are species dependent (Fu et al., 2019).

### 5.2 Mechanisms for correlating reflectance spectra with photosynthetic capacities

Both the reflectance spectra‐based and spectral indices‐based approaches highlighted similar spectral regions (Figures 5, 6, and 10) used in *V*_*cmax*_ and *J*_*max*_ predictions. As shown in Figure 10, PLSR coefficients for predicting *V*_*cmax*_ and *J*_*max*_ were consistent with those at the leaf level (e.g., Ainsworth et al., 2014) and agreed with the current understanding of the correlation between reflectance spectra and foliar biochemistry and physiology (Dechant et al., 2017; Serbin et al., 2015). For example, *V*_*cmax*_ and *J*_*max*_ appear to be strongly associated with reflectance at 700 and 720 nm (the red edge), pointing to the fact that healthy green vegetation absorbs radiation at long red wavelengths. For *V*_*cmax*_ predictions, the wavelength at 768.2 nm was observed as it had a high PLSR coefficient (Figure 10) and correlation coefficient (Figures 5 and 6). This high regression/correlation coefficient may indicate a strong relationship between solar‐induced fluorescence (SIF) and photosynthetic capacities at the canopy level because the SIF signal at ~768 nm can be easily detected with the camera of high spectral resolution (~2.0 nm) used in this study with the spectral fitting method (Zhang et al., 2018; Zhang et al., 2014) and is included in the reflectance spectra for predictions of photosynthetic capacities. Further analysis of the contribution of SIF signals to predictions of photosynthetic variables is still necessary in our future work.

Further examination of correlation or regression coefficients as shown in Figures 5, 6, and 10 suggested that spectral regions associated with leaf characteristics such as structure and pigments also played an important role in *V*_*cmax*_ and *J*_*max*_ predictions. Recently, Dechant et al. (2017) concluded that the prediction of *V*_*cmax*_ and *J*_*max*_ using reflectance spectra for tree species was mainly attributed to the relationship between reflectance spectra and nitrogen content per area. Our results also highlighted important spectral regions at 600 and 660 nm related to nitrogen content (Carter, 1994; Faurtyot & Baret, 1997) that were used for both *V*_*cmax*_ and *J*_*max*_ predictions. A higher nitrogen content per area was generally translated to a greater photosynthetic capacity due to its importance as a component of the enzyme Rubisco (Wright et al., 2004). However, the correlation/regression coefficient in this study did not suggest a dominating factor of nitrogen content to explain photosynthetic variations among crop cultivars, which would probably be explained by the fact that species used in this study included both wild‐type and genetically modified cultivars that may decouple the well‐known relationship between leaf nitrogen and photosynthetic capacities (Meacham‐Hensold et al. 2019).

**Figure 12:**
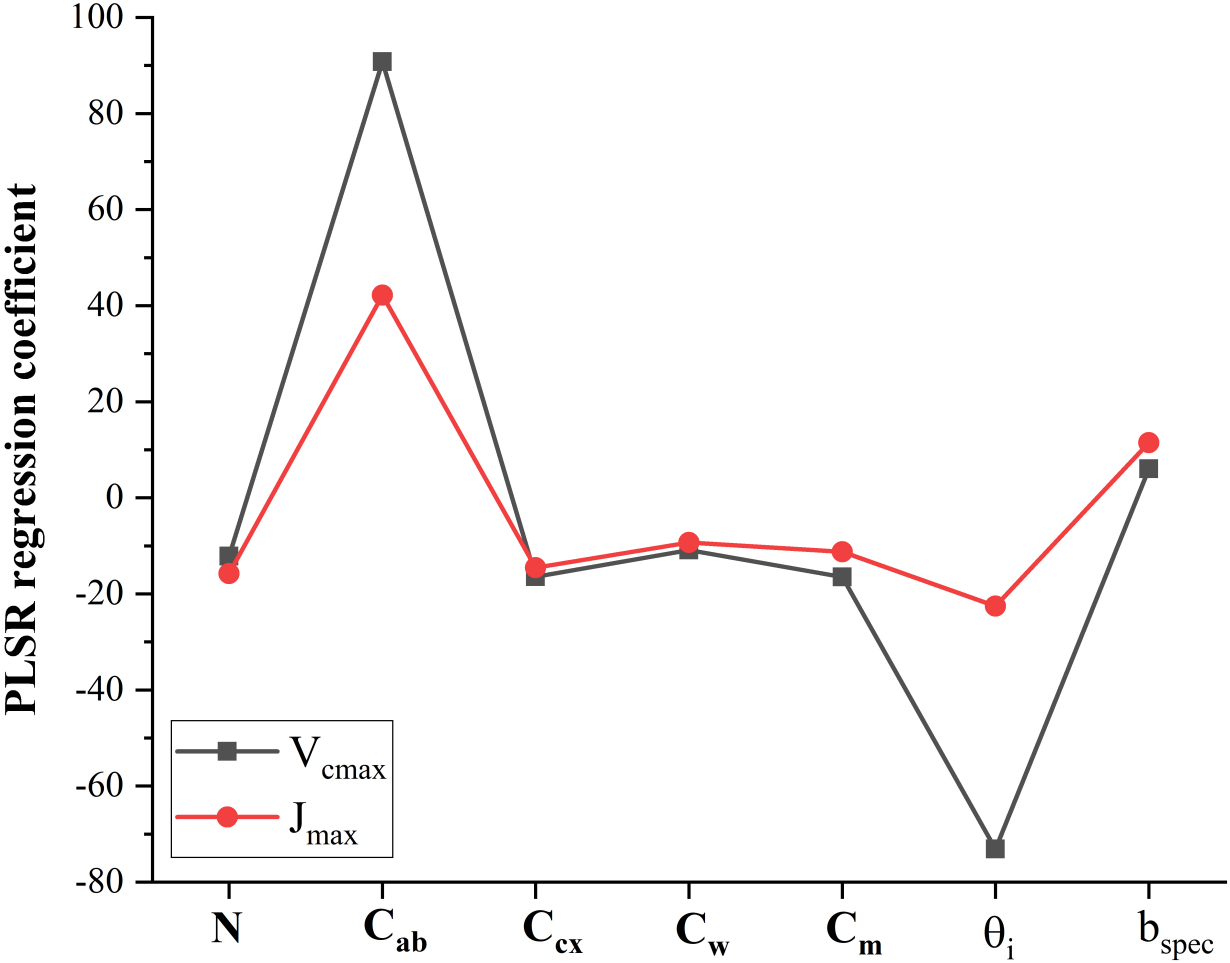
The partial least square regression (PLSR) regression coefficient for the radiative transfer model‐based approach to predict *V*_*cmax*_ and *J*_*max*_. The *x*‐axis refers to the seven parameters derived from reflectance spectra using the PROCOSINE model. Leaf structure parameter *N*, chlorophyll content *C*_*ab*_ (μg/cm^2^), carotenoid content *C*_*cx*_ (μg/cm^2^), equivalent water thickness *C*_*w*_ (cm), dry matter content *C*_*dm*_ (g/cm^2^), light incident angle in degree *θ*_*i*_, and specular parameter *b*_*spec*_ (unitless). The seven variables input to the PLSR were normalized by (raw‐mean)/std so the regression coefficients were comparable

Compared with the other two approaches (as shown in Figures 4 and 7), PLSR based on RTM‐derived parameters had the worst predictive performance. This finding should be reasonable as the reduction of hyperspectral reflectance variables (248 variables from 400 to 900 nm) into only seven parameters through the PROCOSINE model would lose spectral information associated with photosynthetic capacity. Furthermore, prediction errors in these seven leaf parameters existed as the PROCOSINE model assumed negligible diffuse illumination conditions (Jay et al., 2016). However, the RTM‐based approach revealed that important leaf characteristics associated with photosynthesis as the seven parameters derived from reflectance spectral together could explain ~60% of variance (based on the *R* ^2^ in Figure 9) in both *V*_*cmax*_ and *J*_*max*_. More specifically, as shown in Figure 12, the PLSR components used to predict *V*_*cmax*_ and *J*_*max*_ were highly affected by light incident angle (defined as sun zenith angle in Jay et al., 2016; negative regression coefficient) and leaf chlorophyll content (positive regression coefficient). The good relationship between chlorophyll content and photosynthetic capacities has been reported in previous literatures (e.g., Houborg et al., 2013; Croft et al., 2017) because chlorophyll is important in harvesting light for photosynthesis for the reactions of the Calvin‐Benson cycle. In addition, light incident angle was strongly associated with both *V*_*cmax*_ and *J*_*max*_ predictions probably because it was related to the amount of light radiation received by leaves at the canopy level. This implied that *V*_*cmax*_ and *J*_*max*_ predictions were sensitive to the surrounding light environments, which may be tied to changes of nitrogen fractions invested in Rubisco and pigment‐associated proteins (Evans & Poorter, 2001). Despite the possible insights from the RTM‐based approach, caveats should be made for these interpretations because only model‐derived leaf parameters rather than actual measurements were used for analysis. As the PROCOSINE model showed good fitting performance and validation against measured chlorophyll *a + b* content, it was assumed in this study that other six leaf parameters were also estimated with high accuracy.

### 5.3 Implications for broad‐scale mapping of *V_cmax_* and *J*_*max*_ from satellite remote sensing

The current study along with previous studies Fu et al. (2019) and Serbin et al. (2015) provided evidence that the PLSR‐based approach for predicting *V*_*cmax*_ *and J*_*max*_ could be scaled from leaf to plot and to landscape levels. The developed approaches, especially reflectance spectra‐based and spectral indices‐based approaches, can be easily used to map *V*_*cmax*_ and *J*_*max*_ at regional and global scales using satellite hyperspectral images. Future hyperspectral remote sensing missions such as NASA's Surface Biology and Geology mission (National Academies of Sciences, Engineering & Medicine, 2018) and the German Environmental Mapping and Analysis Program (Stuffler et al., 2007) will provide unique potential for deriving spatially and temporally continuous plant physiology information. As the current study was conducted at the plot level using close‐range sensing platforms, it is expected that more considerations should be taken into the provision of readily useable reflectance product from satellite‐based remote sensing platforms in the future. At present, no atmospheric correction was made to the hyperspectral images collected at the plot level. This correction should be implemented for satellite or airborne remote sensing due to stronger atmospheric absorption and scattering that can seriously obscure optical properties of land surfaces. Furthermore, spurious variations in reflectance spectra induced by multisource variability such as plant geometry and architecture, leaf scattering properties, and background soil should be also accounted for at a broader scale using satellite data. Compared with hyperspectral imagery collected from the close‐range platform used in this study, satellite‐based hyperspectral data generally have a much coarse spatial resolution. This coarse spatial resolution at a broad scale may further degrade the modelling performance provided in this study because reflectance spectra from each pixel may contain an ensemble spectral signature of sunlit and shaded leaves from the same or different plant species.

Despite success of the current study, the analysis was limited to wild and genetically modified tobacco cultivars. As such, the scaling of the developed approaches to a broader scale requires further efforts in collecting ground‐truth *V*_*cmax*_ and *J*_*max*_ across a diverse population of plant species within each ecoregion/ecosystem for model calibration and validation. However, the collection of ground‐truth *V*_*cmax*_ and *J*_*max*_ across ecosystems is time consuming and not a trivial matter. Thus, the examination of the developed approaches to estimate *V*_*cmax*_ and *J*_*max*_ at local scale (e.g., small agricultural fields with different plant species) is still necessary to help overcome challenges induced by insufficient sampling of ground‐truth measurements at global scale. For example, Croft et al. (2017) suggested that leaf chlorophyll content, compared with leaf nitrogen content, provided more accurate estimations of *V*_*cmax*_ within a deciduous forest. This conclusion can be further explored in agricultural fields with a more diverse population of plant species. The performance of leaf chlorophyll content as a proxy for *V*_*cmax*_ at agricultural fields may further reveal expectations of performance of leaf chlorophyll content to capture photosynthetic variations at ecosystem level. Finally, at a broad scale, the RTM‐based approach (using PROSAIL rather than PROCOSINE) to predict photosynthetic capacities should be further examined because of large uncertainties of model inversions that may be induced by the LUT approach.

### 5.4 Improving the characterization of photosynthesis in process‐based crop models

The developed approaches to predict photosynthetic capacities for both wild‐type and genetically modified tobacco cultivars from reflectance spectra suggested promising means to improve the characterization of photosynthesis in global process‐based crop models. These crop models, for example, participant models in the Agricultural Model Intercomparison and Improvement Project (Rosenzweig et al., 2014), are the principle ways to understand response of crop yield to climate change factors including temperature, precipitation, and CO_2_ (Bassu et al., 2014; Deryng et al., 2016; Schauberger et al., 2017; Wang et al., 2017). However, photosynthesis representation is generally not explicitly included in these global crop models, limiting the understanding of impacts of enhancing photosynthesis on crop yield under various environmental factors (Wu et al., 2019). Currently, photosynthesis improvements, primarily related to modifications of *V*_*cmax*_, *J*_*max*_, and mesophyll conductance for CO_2_ (von Caemmerer & Evans, 2010; von Caemmerer & Furbank, 2016), are expected as important avenues to increase crop yield to satisfy growing demand for food, fuel, and clothing in the future (Long et al., 2015). It remains a scientific issue whether enhancing leaf photosynthesis would translate to higher crop yield and biomass due to the complex interactions between crop growth and the surrounding environmental factors (Wu et al., 2019). Thus, accurate characterization of photosynthesis and its coupling with global crop models have the potential to assess and guide photosynthetic manipulation efforts. As hyperspectral remote sensing data become available in the future, the developed approaches have the potential to yield global photosynthetic parameters that can be input to the crop models to understand the impacts of photosynthetic improvements on crop yield under various climate conditions. Further work is needed to possibly include a data assimilation module within the global crop models to take in photosynthetic information directly rather than to use a predefined relationship between photosynthetic parameters and specific leaf nitrogen (De Pury & Farquhar, 1997). This predefined empirical relationship may not be able to fully capture variations in photosynthesis to account for crop yield variability in both spatial and temporal domains.

## Supporting information


**Appendix S1** Supporting Information

**Table S1** The ground‐truth *V*_*cmax*_ and *J*_*max*_ and the corresponding column number of the reflectance spectra in the data matrix (48 rows by 240 columns provided in the “plot‐level reflectance spectra.xlsx”). The *J*_*max*_ column missed some data values as the corresponding cultivars are not electron transport limited.
**Data S1** The processed reflectance spectra at plot level, ground‐truth *V*_*cmax*_ and *J*_*max*_, and the reflectance based PLS regression coefficients for *V*_*cmax*_ and *J*_*max*_ are available in Supporting Information.
