# Supplementary material for: Estimating photosynthetic traits from reflectance spectra: A synthesis of spectral indices, numerical inversion, and partial least square regression
Source: Plant Cell Environ. 2020 Feb 27;43(5):1241–58. doi: 10.1111/pce.13718 (PMC7385704; doi:10.1111/pce.13718)
Supplement: Supplementary file 2 — Table S1 The ground‐truth V cmax and J max and the corresponding column number of the reflectance spectra in the data matrix (48 rows by 240 columns provided in the “plot‐level reflectance spectra.xlsx”). The J max column missed some data values as the corresponding cultivars are not electron transport limited. Data S1 The processed reflectance spectra at plot level, ground‐truth V cmax and J max, and the reflectance based PLS regression coefficients for V cmax and J max are available in Supporting Information. [file PCE-43-1241-s002.docx]

**Supporting Information**

**(1)** Plot Level Reflectance Spectra: 48 plots in total. The data excel (“Plot level reflectance spectra.xlsx”) was organized as a 48 (row, i.e., observations) by 240 (column, i.e., wavelength) by matrix. The matrix row follows the order as specified in Table S1 in which the ground-truth *V_cmax_* and *J_max_* can also be found.

**(2)** Reflectance based PLSR coefficients: The reflectance based PLSR coefficients were provided in “*Reflectance based PLSR coefficients for Vcmax.txt*” and “*Reflectance based PLSR coefficients for Jmax*.txt”.

**Table S1**. The ground-truth *V_cmax_* and *J_max_* and the corresponding column number of the reflectance spectra in the data matrix (48 rows by 240 columns provided in the “plot-level reflectance spectra.xlsx”). The *J_max_* column missed some data values as the corresponding cultivars are not electron transport limited.

| Row Order | Date | Plot ID | Repetitions | *V_cmax_* | *J_max_* | Year | Cultivar ID by number |
| --- | --- | --- | --- | --- | --- | --- | --- |
| 1 | 6/22/2017 | PH | 1 | 162.4379 | 208.2598 | 2017 | 1 |
| 2 | 6/22/2017 | PH | 2 | 191.5860 | 234.2137 | 2017 | 1 |
| 3 | 6/22/2017 | PH | 3 | 196.1954 | 238.4901 | 2017 | 1 |
| 4 | 6/22/2017 | PH | 4 | 202.7483 | 238.1527 | 2017 | 1 |
| 5 | 6/26/2017 | 200-8 | 1 | 131.0665 | 185.2944 | 2017 | 5 |
| 6 | 6/26/2017 | 200-8 | 2 | 124.7260 | 174.9333 | 2017 | 5 |
| 7 | 6/27/2017 | 200-8 | 3 | 141.9415 | 188.9038 | 2017 | 5 |
| 8 | 6/27/2017 | 4-KO | 2 | 156.0183 | 194.5969 | 2017 | 6 |
| 9 | 6/27/2017 | 4-KO | 3 | 210.2018 | 235.9975 | 2017 | 6 |
| 10 | 6/27/2017 | 4-KO | 4 | 170.8086 | 229.3717 | 2017 | 6 |
| 11 | 6/27/2017 | 43-OE | 1 | 131.6151 | 170.9038 | 2017 | 7 |
| 12 | 6/27/2017 | 43-OE | 3 | 178.3458 | 223.1465 | 2017 | 7 |
| 13 | 7/6/2018 | Ssus | 1 | 223.3103 | 292.9435 | 2017 | 4 |
| 14 | 7/6/2018 | Ssus | 2 | 214.2412 | 292.0100 | 2017 | 4 |
| 15 | 7/6/2018 | Ssus | 3 | 206.5660 | 271.3354 | 2017 | 4 |
| 16 | 7/6/2018 | Ssus | 4 | 209.4329 | 291.3877 | 2017 | 4 |
| 17 | 7/7/2017 | Samsun | 1 | 298.2764 | 298.4138 | 2017 | 2 |
| 18 | 7/7/2017 | Samsun | 2 | 270.1162 | 321.6735 | 2017 | 2 |
| 19 | 7/7/2017 | Samsun | 3 | 316.3633 | 338.7081 | 2017 | 2 |
| 20 | 7/7/2017 | Samsun | 4 | 318.9624 | 327.3909 | 2017 | 2 |
| 21 | 7/12/2017 | Mammoth | 1 | 262.4501 | 274.6534 | 2017 | 3 |
| 22 | 7/12/2017 | Mammoth | 2 | 298.3135 | 279.5103 | 2017 | 3 |
| 23 | 7/12/2017 | Mammoth | 3 | 285.7849 | 302.4000 | 2017 | 3 |
| 24 | 7/12/2017 | Ssud | 1 | 62.6418 |  | 2017 | 11 |
| 25 | 7/12/2017 | Ssud | 2 | 76.3991 |  | 2017 | 11 |
| 26 | 7/12/2017 | Ssud | 3 | 78.9117 |  | 2017 | 11 |
| 27 | 7/31/2018 | Ssud | 1 | 46.1002 |  | 2017 | 11 |
| 28 | 7/31/2018 | Ssud | 2 | 35.9805 |  | 2017 | 11 |
| 29 | 7/31/2018 | Ssud | 3 | 31.7696 |  | 2017 | 11 |
| 30 | 7/31/2018 | Ssud | 4 | 15.9840 |  | 2017 | 11 |
| 31 | 7/31/2018 | Ssus | 2 | 225.7435 | 282.9695 | 2017 | 4 |
| 32 | 7/31/2018 | Ssus | 3 | 192.2938 | 269.9270 | 2017 | 4 |
| 33 | 8/18/2017 | Mammoth | 1 | 186.0596 | 239.7669 | 2017 | 3 |
| 34 | 8/18/2017 | Mammoth | 2 | 251.2258 | 273.7279 | 2017 | 3 |
| 35 | 8/18/2017 | Mammoth | 3 | 163.4987 | 240.8567 | 2017 | 3 |
| 36 | 8/18/2017 | Samsun | 1 | 314.0576 | 317.2558 | 2017 | 2 |
| 37 | 8/18/2017 | Samsun | 2 | 177.2961 | 228.4586 | 2017 | 2 |
| 38 | 8/18/2017 | Samsun | 3 | 176.9717 | 217.4578 | 2017 | 2 |
| 39 | 7/24/2018 | PH | 2 | 150.6182 | 215.1079 | 2018 | 1 |
| 40 | 7/24/2018 | PH | 4 | 271.6485 | 282.5629 | 2018 | 1 |
| 41 | 7/24/2018 | Samsun | 4 | 287.7779 | 281.6613 | 2018 | 2 |
| 42 | 7/24/2018 | Ssud | 4 | 24.2144 |  | 2018 | 11 |
| 43 | 7/24/2018 | VPZ-23 | 4 | 173.3014 | 238.6752 | 2018 | 8 |
| 44 | 7/25/2018 | 200-8 | 3 | 125.8903 | 160.1918 | 2018 | 5 |
| 45 | 7/25/2018 | 201-8 | 4 | 185.3408 | 238.8421 | 2018 | 9 |
| 46 | 7/25/2018 | 423-16 | 3 | 186.4126 | 266.6705 | 2018 | 10 |
| 47 | 7/25/2018 | 43-OE | 4 | 109.4671 | 118.8519 | 2018 | 7 |
| 48 | 7/25/2018 | Ssud | 2 | 18.9049 |  | 2018 | 11 |
